# Immature Dentate Gyrus: An Endophenotype of Neuropsychiatric Disorders

**DOI:** 10.1155/2013/318596

**Published:** 2013-06-12

**Authors:** Hideo Hagihara, Keizo Takao, Noah M. Walton, Mitsuyuki Matsumoto, Tsuyoshi Miyakawa

**Affiliations:** ^1^Division of Systems Medical Science, Institute for Comprehensive Medical Science, Fujita Health University, 1-98 Dengakugakubo, Kutsukake-cho, Toyoake, Aichi 470-1192, Japan; ^2^CREST, Japan Science and Technology Agency, 4-1-8 Honcho, Kawaguchi, Saitama 332-0012, Japan; ^3^Section of Behavior Patterns, Center for Genetic Analysis of Behavior, National Institute for Physiological Sciences, 5-1 Aza-Higashiyama, Myodaiji-cho, Okazaki, Aichi 444-8787, Japan; ^4^CNS, Astellas Research Institute of America LLC, 8045 Lamon Avenue, Skokie, IL 60077, USA

## Abstract

Adequate maturation of neurons and their integration into the hippocampal circuit is crucial for normal cognitive function and emotional behavior, and disruption of this process could cause disturbances in mental health. Previous reports have shown that mice heterozygous for a null mutation in **α**-CaMKII, which encodes a key synaptic plasticity molecule, display abnormal behaviors related to schizophrenia and other psychiatric disorders. In these mutants, almost all neurons in the dentate gyrus are arrested at a pseudoimmature state at the molecular and electrophysiological levels, a phenomenon defined as “immature dentate gyrus (iDG).” To date, the iDG phenotype and shared behavioral abnormalities (including working memory deficit and hyperlocomotor activity) have been discovered in Schnurri-2 knockout, mutant SNAP-25 knock-in, and forebrain-specific calcineurin knockout mice. In addition, both chronic fluoxetine treatment and pilocarpine-induced seizures reverse the neuronal maturation, resulting in the iDG phenotype in wild-type mice. Importantly, an iDG-like phenomenon was observed in post-mortem analysis of brains from patients with schizophrenia/bipolar disorder. Based on these observations, we proposed that the iDG is a potential endophenotype shared by certain types of neuropsychiatric disorders. This review summarizes recent data describing this phenotype and discusses the data's potential implication in elucidating the pathophysiology of neuropsychiatric disorders.

## 1. Introduction

The exact mechanisms within the brain that underlie most psychiatric disorders remain largely unknown, and one of the major challenges in psychiatric research is to identify the pathophysiology in the brains of patients with these disorders. This is challenging because psychiatric disorders are diagnosed on the basis of behavioral characteristics and not biological criteria. Therefore, each psychiatric disorder likely consists of multiple biologically heterogeneous populations, which further complicates the search for underlying pathophysiologies. 

Previously, studies have identified the “immature dentate gyrus (iDG),” a potential brain endophenotype shared by several psychiatric disorders, including schizophrenia and bipolar disorder. The iDG was identified in animal models of psychiatric disorders, which were selected using large-scale behavioral screening of genetically engineered mice [1]. In the iDG phenotype, most of the granule cells or principal neurons in the dentate gyrus (DG) within the hippocampus are arrested at a pseudoimmature status, in which the molecular and physiological properties are similar to those of normal immature neurons from the DG. To date, the iDG phenotype has been identified in several strains of mutant mice, including *α*-CaMKII heterozygous knockout (HKO) mice [1], Schnurri-2 (Shn-2) knockout (KO) mice [2], mutant SNAP-25 knock-in (KI) mice [3], and forebrain-specific calcineurin (CN) KO mice [4]. A signature pattern, quite similar to the iDG phenotype identified in these mutant mice, has also been found in mice treated with chronic fluoxetine [5] and in a pilocarpine-induced mouse model of epilepsy [6]. Moreover, postmortem analysis for molecular markers of neuronal maturity in the DG revealed an iDG-like signature for brains of patients with schizophrenia and bipolar disorder [7].

The purpose of this review is to introduce how the iDG phenotype was identified and its molecular and cellular signature. An additional goal is to summarize potential mechanisms underlying the iDG phenotype and its impact on brain functions.

## 2. Discovery of iDG in ***α*-**CaMKII HKO Mice

**Figure 1:**
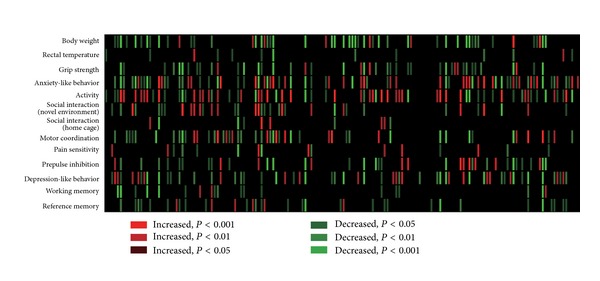
Heat map showing behavioral phenotypes of more than 160 strains of genetically engineered mice. Each column represents the mouse strain analyzed in the laboratory of the author's group (unpublished data). Each row represents a behavior category assessed by the comprehensive battery of behavioral tests. Colors represent an increase (red) or decrease (green), compared between wild-type and mutant mice. Adapted from Takao et al. [8].

A high-throughput comprehensive behavioral testing battery was developed for many different strains of genetically engineered mice. The battery of tests covers many behavioral domains ranging from sensorimotor functions to highly cognitive functions, like learning and memory. In the course of a large-scale effort to phenotype the genetically engineered mice (mutant mice), this battery of tests was administered to more than 160 strains of knockout and transgenic mice (Figure 1) [8]. Surprisingly, when tested most of the strains of mutant mice displayed at least some aberrant behavioral phenotypes, which may suggest that many of the genes expressed in the brain may have some functional significance at the behavior level. In the course of screening the mutant mice populations, a number of mouse strains were identified that showed abnormal behaviors common to neuropsychiatric disorders (e.g., schizophrenia, bipolar disorder, and autism). Among these mutant mouse models, the *α*-CaMKII HKO mice showed a particularly dramatic series of abnormal behaviors. The first discovery of the iDG phenotype occurred during investigations into the neuropathophysiology underlying these behaviors.

Calmodulin-dependent protein kinase II (CaMKII) is a major downstream molecule of the *N*-methyl-d-aspartic acid (NMDA) receptor, which has presumed involvement in the pathophysiology of schizophrenia. The function of the NMDA receptor can be modulated by calcineurin, a potential susceptibility gene we previously identified [9, 10]. CaMKII is thought to play an essential role in neural plasticity, such as long-term potentiation and long-term depression [11].

**Figure 2:**
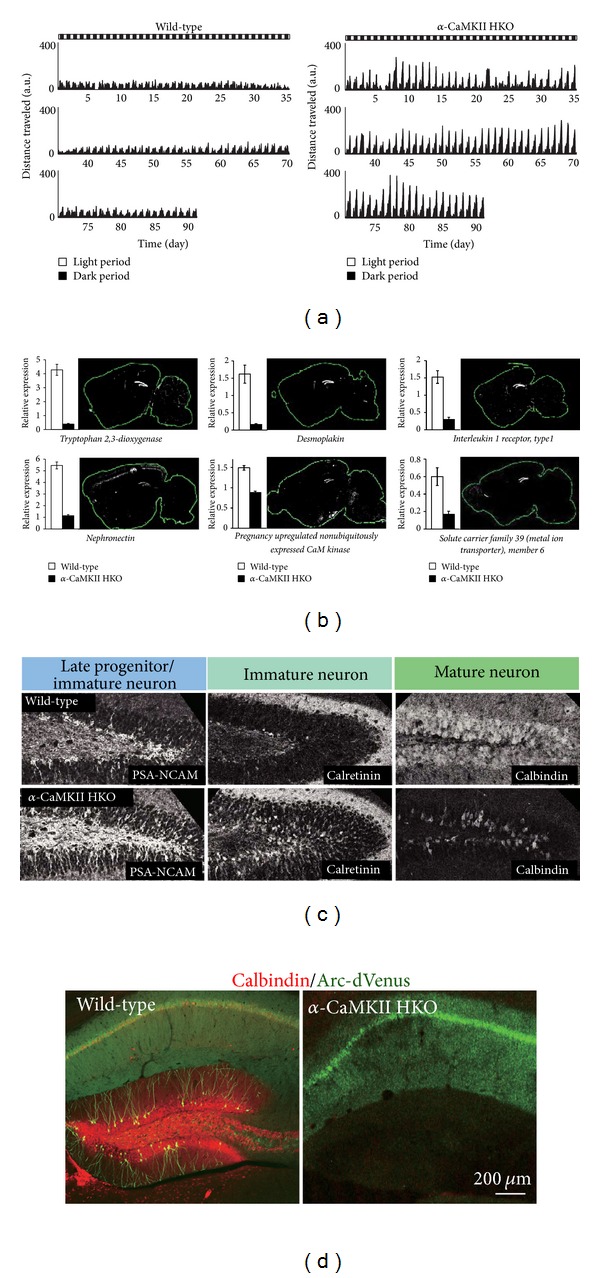
Mood-change-like behavior and the iDG phenotype in *α*-CaMKII HKO mice. (a) Pattern of locomotor activity of wild-type and *α*-CaMKII HKO mice in their home cage. Mutant mice were hyperactive and showed a periodic mood-change-like activity pattern. A.U: arbitrary unit. (b) Among the downregulated genes in the hippocampus of *α*-CaMKII HKO mice, several genes were expressed selectively in the DG (Allen Brain Atlas [42]. Seattle, WA: Allen Institute for Brain Science. © 2004–2008. Available from: http://www.brain-map.org). Graphs indicate the relative expression levels of each gene in the microarray experiment. (c) In *α*-CaMKII HKO mice, expression of PSA-NCAM (a late-progenitor and immature-neuron marker) and calretinin (an immature neuron marker) was markedly increased, and expression of calbindin (a mature neuron marker) was decreased. (d) Expression of Arc-dVenus in the DG of *α*-CaMKII HKO mice after the working memory task (eight-arm radial maze test) was completely abolished. *Red:* calbindin; *green:* Arc-dVenus. Adapted from Yamasaki et al. [1] and Matsuo et al. [13].

Perhaps the most striking behavioral phenotype of *α*-CaMKII HKO mice is that they occasionally kill their cage mates (often littermates). Prior to 6 months of age, *α*-CaMKII HKO mice kill more than half of their group-housed cage mates. In addition, these mice show greatly increased locomotor activity in the open field test. While *α*-CaMKII HKO mice demonstrate normal reference memory, as assessed by the reference memory version of the eight-arm radial maze test, their working memory is severely impaired. Likewise, in the T-maze forced-alternation test, the working memory of the *α*-CaMKII HKO mice was found to be severely impaired, while they showed normal performance in a simple left/right discrimination task using the same apparatuses. Previous studies have presented representative videos of the performance of *α*-CaMKII HKO mice in these tasks [12]. Using the home cage locomotor activity monitoring system, which measures distance travelled in the home cage, it was shown that the locomotor activity patterns of the *α*-CaMKII HKO mice slowly and dramatically change over time. These mice show a periodic mood-change-like behavior in their home cage (Figure 2(a)), and 1 cycle lasts approximately 10–20 d. The manifestations of this behavioral phenotype are so clear that these mutant mice can be easily identified using these activity patterns.

The next task was to understand the underlying pathophysiology in the brains of the *α*-CaMKII mutants that show this dramatic array of behavioral abnormalities. First, a transcriptome analysis of the hippocampus of mutant mice was conducted. Gene expression in *α*-CaMKII HKO mice was highly dysregulated, with altered expressions of more than 2,000 genes in the hippocampus of the mutants. Surprisingly, of the top 30 hippocampal genes with downregulated expression in *α*-CaMKII mutants, 6 genes showed highly selective expression in the DG (Figure 2(b)). These data implied the existence of some unknown abnormalities in the DG of these mutants. 

Adult neurogenesis was then examined and a large upregulation (>50%) of neurogenesis was demonstrated in *α*-CaMKII mutant mice. Traditionally, the process of adult neurogenesis involves a recapitulation during which stem/progenitor cells give way to immature postmitotic neurons, which then mature to adopt distinct morphological and physiological properties in the hippocampus. Based on the observed upregulation of adult neurogenesis in the *α*-CaMKII mutant mice, it was interesting to determine whether the *α*-CaMKII mutant mice followed the traditional path of neurogenesis. Gene chip analysis of hippocampus demonstrated a reduced expression of the gene for calbindin, a marker for mature neurons in the DG, in the mutants. Furthermore, calbindin, as assessed by immunohistochemistry, was dramatically reduced in the DG of the *α*-CaMKII mutants (Figure 2(c)). In addition, expression of calretinin, a marker for immature neurons in the DG, was increased, as was expression of polysialylated neuronal cell adhesion molecule (PSA-NCAM), a marker for late progenitor cells and immature neurons. Taken together, these findings suggest that in *α*-CaMKII mutant mice the number of immature neurons are upregulated and the number of mature neurons are downregulated. 

Golgi staining was also performed to examine the morphology of the DG neurons. Interestingly, staining of the DG was difficult to achieve in the *α*-CaMKII mutant mice, presenting a challenge in the quantification of morphological alterations. Fewer dendritic intersections and shorter overall dendritic length were demonstrated in the *α*-CaMKII mutant mice, consistent with the idea that DG neurons in these mice are immature.

Moreover, DG neurons in the *α*-CaMKII mutant mice were found to have many electrophysiological features that are known characteristics of immature dentate neurons, including high input resistance and a decreased number of spikes during sustained depolarization. This finding indicates that, in the *α*-CaMKII mutant mice, most of the DG neurons have electrophysiological properties consistent with those of immature neurons. Interestingly, *α*-CaMKII HKO mice also exhibit abnormal synaptic transmission/plasticity at mossy fiber-CA3 synapses. Specifically, *α*-CaMKII mutant mice have increased basal transmission and dramatically decreased facilitation at these synapses. 

The *α*-CaMKII HKO mice were mated with transgenic mice expressing destabilized Venus (dVenus) under the Arc promoter. Arc is known to be induced by neural activity. In normal mice, a number of hippocampal neurons (including DG neurons) become positive for Arc-dVenus after performing working memory tasks (Figure 2(d)). In *α*-CaMKII HKO mice, Arc-dVenus expression is almost completely abolished in the DG after performing a working memory task, suggesting that the DG in these mutants is not fully functional [13].

These experiments were the first to demonstrate that the most of the DG neurons in *α*-CaMKII HKO mice are arrested at a pseudoimmature status. This arrest of maturity was termed the “immature dentate gyrus (iDG)” phenotype. 

## 3. iDG Is an Endophenotype Shared by Genetically Engineered Mice with Abnormal Behaviors Characteristic for Neuropsychiatric Disorders

After identification of the iDG phenotype, it was important to investigate whether other strains of mutant mice share this phenotype. Over the past 10 years, the behaviors of mutant mice have been assessed continuously using the comprehensive behavioral test battery previously described [8]. The majority of the behavioral phenotypes reported are registered in the mouse phonotype database (http://www.mousephenotype.org). This database was used to identify strains of mice that exhibited behavioral phenotype(s) similar to that shown by the *α*-CaMKII mutants. More than 160 different strains of mutant mice were investigated, and the best behavioral match was identified in a strain of the mice lacking a transcription factor called Schnurri-2 (Shn-2 KO mice) [2].

**Figure 3:**
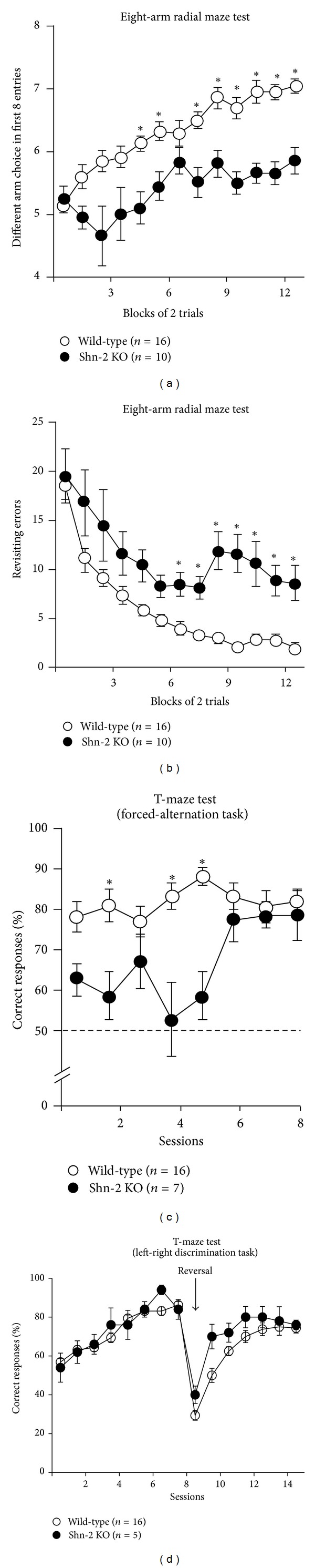
Impaired working memory performance in Shn-2 KO mice. (a) In the spatial working memory version of the eight-arm radial maze, compared to controls, Shn-2 KO mice performed significantly worse with respect to the number of different arm choices in the first 8 entries (genotype effect: F_1,24_ = 62.104, *P* < 0.0001). (b) Mutants made significantly more revisiting errors than controls (genotype effect: F_1,24_ = 45.597, *P* < 0.0001; genotype × trial block interaction: F_12,228_ = 1.470, *P* = 0.1345). (c) Shn-2 KO mice also showed poor working memory performance in the T-maze forced-alternation task (genotype effect: F_1,21_ = 20.497: *P* = 0.0002; genotype × session interaction: F_7,147_ = 3.273: *P* = 0.0029). (d) Shn-2 KO and wild-type mice were comparable in the left-right discrimination task (genotype effect: *F*_1,19_ = 0.209,  *P* = 0.6529) and reversal learning (genotype effect: *F*_1,19_ = 5.917,  *P* = 0.0251). Asterisks indicate statistical significance determined using the Student's *t*-test with a correction for multiple comparisons in each block (a, b) and each session (c) (*P < 0.05). Adapted from Takao et al. [2].

Shn-2 KO mice show an array of behavioral abnormalities that is quite similar to those of *α*-CaMKII HKO mice, including hyperlocomotor activity and severe deficits in working memory. In Shn-2 KO mice, locomotor activity is dramatically increased and prepulse inhibition (PPI) is impaired [2]. However, Shn-2 KO mice have normal reference memory, as assessed by the left-right discrimination task performed in a T-maze. In addition, the Shn-2 KO mice have severely impaired working memory, as suggested by their performance in the T-maze forced-alternation task and the eight-arm radial maze (Figures 3(a)–3(d)) [2].

**Figure 4:**
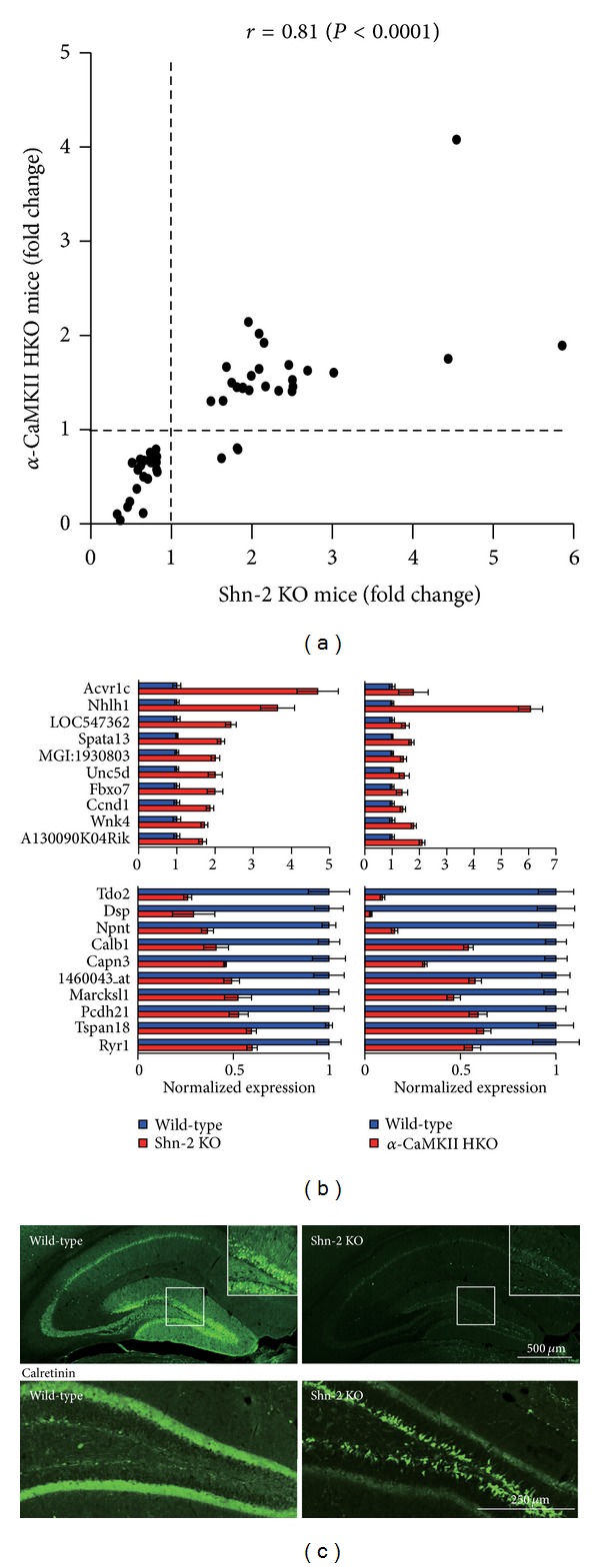
Maturation abnormalities in DG neurons of Shn-2 KO mice. (a) The hippocampal transcriptome pattern of Shn-2 KO mice is similar to that of *α*-CaMKII HKO mice, which also demonstrated maturation abnormalities in the DG. Genes showing differential expression between genotypes at *P* < 0.005 in both experiments were plotted. (b) Normalized gene expression of differentially expressed genes in Shn-2 KO and *α*-CaMKII HKO mice. The top 10 genes are indicated in the graphs. (c) Expression of the mature neuronal marker calbindin was decreased, and the expression of the immature neuronal marker calretinin was markedly increased in the DG of Shn-2 KO mice. Adapted from Takao et al. [2].

To identify the pathophysiologic abnormalities responsible for the behavioral phenotypes in Shn-2 KO mice, an additional gene chip analysis was conducted and the hippocampal transcriptome patterns of Shn-2 KO mice were compared to those of *α*-CaMKII HKO mice. Remarkably, the 2 mutant strains showed strikingly similar gene expression patterns in the hippocampus (Figures 4(a) and 4(b)), with expression of >100 genes altered in a similar manner with regard to both the direction and the magnitude of the alterations. For example, as observed in *α*-CaMKII HKO mice, calbindin expression was dramatically decreased in the DG of Shn-2 KO mice (Figure 4(c)) [2]. Whole-cell patch recordings made from granule cells in the DG revealed that DG neurons in Shn-2 KO mice have electrophysiological features shown by immature neurons, such as a lower current threshold for firing, a short latency to the first spike, and a decreased number of spikes during sustained depolarization [2]. This evidence supports the conclusion that Shn-2 KO mice also express iDG phenotype. Additional strains of mutant mice with the iDG phenotype have also been identified. 

Synaptosomal-associated protein of 25 kDa (SNAP-25) regulates exocytosis of neurotransmitters and is thought to be involved in the neuropsychiatric disorders such as schizophrenia [14, 15], attention-deficit/hyperactivity disorder (AD/HD) [16, 17], and epilepsy [18, 19]. The iDG phenotype was also identified in SNAP-25 knock-in (KI) mice with a single amino acid substitution [3]. Behaviorally, these mice also show severe deficits in working memory. Of note, chronic administration of valproic acid, an antiepileptic drug, rescued both the iDG phenotype and working memory deficit in SNAP-25 KI mice. CN is a key signal transduction molecule in the brain, and several studies have suggested a link between dysfunction of CN signaling and schizophrenia [9, 20]. Conditional forebrain-specific CN KO mice also exhibit abnormal behaviors related to schizophrenia [10, 21], including a severe working memory deficit, and an iDG phenotype was also observed in CN KO mice [4].

**Figure 5:**
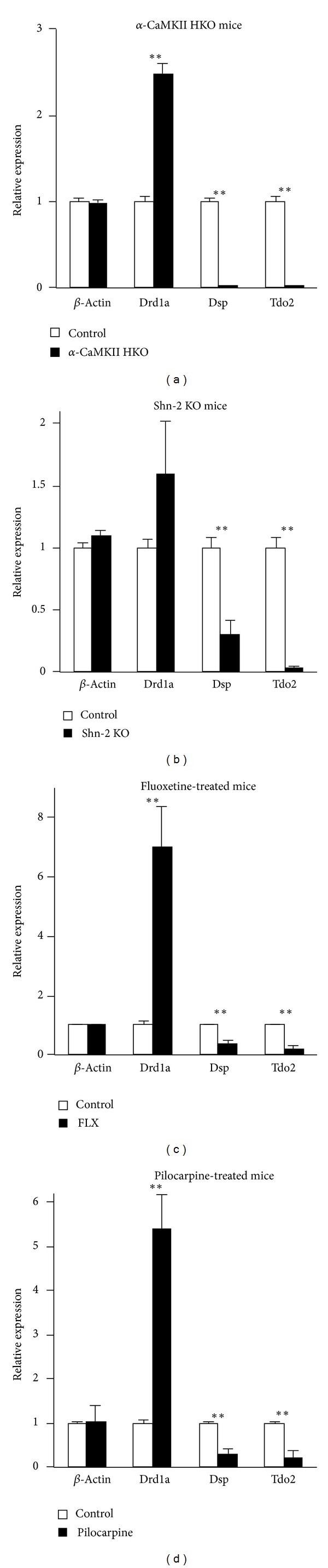
Identification of the iDG phenotype using real-time polymerase chain reaction. iDG is characterized by upregulation of *dopamine receptor D1a* (*Drd1a*) and downregulation of both *desmoplakin* (*Dsp*) and *tryptophan 2,3-dioxygenase* (*Tdo2*) in the hippocampus. Asterisks indicate statistical significance determined using the Student's *t*-test (**P < 0.01, *n* = 4–7 per group). Adapted from Yamasaki et al. [1], Takao et al. [2], Kobayashi et al. [5], and Shin et al. [6].

Further studies have established a method for assessing the existence of the iDG phenotype using real-time polymerase chain reaction (PCR) with 3 probes (Figure 5) [22] selected using gene chip data from *α*-CaMKII HKO mice. The molecular markers for iDG include increased expression of dopamine receptor D1A (*Drd1a*) and decreased expression of tryptophan 2,3-dioxygenase (*Tdo2*) and desmoplakin (*Dsp*) in the DG of mutant mice [1]. To date, based on these molecular markers for iDG, 9 of the 16 strains of mice that exhibited abnormal behaviors (e.g., hyperlocomotor activity, reduced anxiety-like behavior, and impaired working memory) have been shown to express the iDG phenotype. These results suggest that iDG is a common endophenotype shared by mice with behavioral abnormalities characteristic for neuropsychiatric disorders.

Furthermore, the current results demonstrated that hippocampal gene expression patterns of mice expressing a constitutively active cAMP-response element-binding protein (CREB) variant (VP16-CREB) were similar to those of *α*-CaMKII HKO, Shn-2 KO, or SNAP-25 KI mice. Notably, VP16-CREB mice exhibited dramatic alteration in expression of *Calb1*, *Dsp*, glial fibrillary acidic protein (*GFAP*), and complement genes in the hippocampus [23]. It has been shown previously that CREB activation is observed in immature granule cells and plays important roles in differentiation, survival, and maturation of neurons [24]. Thus, it is possible that chronic activation of CREB induced a maturation abnormality of the granule cells, resulting in an iDG-like phenotype.

## 4. Induction of iDG in Wild-Type Animals

The iDG phenotype can be seen not only in genetically engineered mice, but also in properly manipulated wild-type normal mice. For example, Kobayashi et al. found that chronic treatment with the antidepressant fluoxetine (FLX), one of the most widely used selective serotonin reuptake inhibitors (SSRI), can induce iDG phenotypes in adult mice [5]. In FLX-treated mice, expression of *Calb1*, *Tdo2*, and *Dsp*, markers for mature granule cells, was greatly reduced in the DG. In contrast, expression of calretinin, a marker for immature granule cells, was increased in the DG [5]. These findings suggest that the number of immature granule cells is upregulated and the number of mature granule cells is downregulated in FLX-treated mice. Electrophysiologically, FLX-treated granule cells showed higher excitability, which is a functional characteristic observed in immature granule cells. FLX treatment was also found to reduce mossy fiber synaptic facilitation to juvenile levels [5]. These molecular and electrophysiological phenotypes found in FLX-treated mice are strikingly similar to those found in *α*-CaMKII HKO, Shn-2 KO, and SNAP-25 KI mice (see Table 1). This likely represents an example of “dematuration,” in which mature neurons are reversed to a pseudoimmature status. In the study by Kobayashi et al., the dose of FLX used to treat wild-type mice was higher than the doses used in other studies using both wild-type mice [25, 26] and mouse model of depression [27, 28]. This FLX-induced iDG caused by “dematuration” may be related to the antidepressant-induced mania/psychosis observed in clinical settings.

Recently, a second group reported that FLX treatment induced an immature state in basolateral amygdala neurons in adult brains [36]. In their study, chronic FLX treatment reduced the percentage of neurons containing perineuronal nets (PNNs) and expressing parvalbumin (PV) in the region. PNNs predominantly surround mature PV-positive fast-spiking (FS) neurons. PNNs develop late in postnatal development and their formation coincides with the closure of critical developmental periods and the maturation of PV-positive neurons [37, 38]. During the critical period, absence of PNNs is thought to allow the induction of synaptic plasticity. Degradation of PNNs could reactivate synaptic plasticity in adult brain [39, 40]. Thus, FLX treatment shifted the PV- and PNN-containing neurons toward an immature state in the adult basolateral amygdala [36]. The amygdala plays a crucial role in fear-related behaviors. Chronic FLX treatment has been thought to increase synaptic plasticity, converting fear memory circuitry to a more immature state, subsequently allowing erasure of that fear memory. A bidirectional regulation of neuronal maturation in both the DG and amygdala could be an important mechanism of FLX action. In terms of regulation of neuronal maturation, chronic FLX treatment has also been shown to reactivate ocular dominance plasticity in the adult visual cortex [41]. Considering that ocular dominance plasticity is restricted to a critical period during postnatal development [39], chronic FLX treatment could reinstate a juvenile-like form of that plasticity in adulthood. These findings suggest that “dematuration” of neurons caused by FLX is not specific to the DG granule cells.

In addition to FLX, another SSRI, paroxetine, is most likely to induce “dematuration” of DG granule cells in adult mice. Chronic paroxetine treatment has been found to suppress calbindin and *Dsp* expression in the DG [5, 31] and reduce frequency facilitation at mossy fiber-CA3 synapses (mossy fiber synapses formed by immature granule cells exhibit smaller frequency facilitation) [5]. These results suggest that serotonergic antidepressants can reverse the state of neuronal maturation in the adult DG.

Individuals with epilepsy are at significant risk of psychosis with emerging behavioral features that include hallucinations, mood instability, mixed irritability, and mania. Recent genetic studies have shown that chromosome copy number variations, such as the 15q13.3 microdeletion, as conferring an increased risk for developing psychosis and epilepsy and suggest that common phenotypes in epilepsy and psychosis may share a genetic basis. 

Pilocarpine-induced seizure is a well-established rodent model of temporal lobe epilepsy. Based on previous evidence demonstrating a permanent reduction in calbindin mRNA and protein expression from the hippocampus of rats following pilocarpine treatment (and a similar reduction in human epileptic brains post mortem), it was hypothesized that mice with pilocarpine-induced seizures also exhibit an iDG phenotype. Establishment of the iDG phenotype in this mouse model of epilepsy was expected to further support the link between the pathophysiologies of epilepsy and psychosis. Molecular markers for the iDG phenotype were demonstrated in mice with pilocarpine-induced seizures, including dysregulated gene/protein expression of calretinin/calbindin, as well as hallmark alterations in other iDG markers such as *Drd1a, Tdo2*, and *Dsp*. Mice with pilocarpine-induced seizures also displayed characteristic iDG phenotypes at the electrophysiological levels, including decreased polarization of resting membrane potentials, lower spike threshold currents in their DG granule cells [6]. Although these characteristics resemble those found in *α*-CaMKII HKO, DG granule cells in pilocarpine-treated mice exhibited a significant increase in repetitive spiking by sustained depolarizing currents. This was in sharp contrast to the *α*-CaMKII HKO, which showed relatively few spikes following the same stimulation protocol. Granule cells from pilocarpine-treated mice resemble neurons observed in chronic FLX-treated mice. Therefore, similar to what was observed as a result of pilocarpine treatment in mice, seizure-induced changes in the epileptic brain may lead to a “dematuration” of DG granule cells resulting in an iDG [6]. Remarkably, the behavioral phenotypes of mice with pilocarpine-induced seizures are similar to those found in earlier iDG mouse models, specifically hyperlocomotor activity, working memory deficits, and social withdrawal. 

## 5. iDG Phenotype and Human Psychiatric Disorders

The discovery of an iDG phenotype in multiple strains of mice with behavioral abnormalities suggests a link between iDG and the distinct behavioral traits characteristic of schizophrenia and other psychiatric disorders. To assess whether iDG is related to human psychiatric disorders, comprehensive gene expression data from 166 hippocampi were analyzed from human brains postmortem, including brains of 21 patients with psychiatric disorders. Comprehensive gene expression analysis was performed using a specific biomarker set derived from *α*-CaMKII HKO mice, including 10 genes selected (*ADCY8*, *CCND1*, *LOC151835*, *LOC284018*, *NTNG1*, *PDYN*, *PIP3-E*, *PNCK*, *SPATA13,* and *TDO2*) due to differential expression in the iDG mutant hippocampus. Statistical clustering of the gene expression data was performed using all 166 hippocampi from human brains collected postmortem. The analysis roughly classified subjects into 2 clusters, one of which (schizophrenia-enriched cluster) contained 19 of the 21 patients with psychiatric disorders (including schizophrenia, schizoaffective, and bipolar patients) [1]. Furthermore, the gene expression profile was compared between the patients with schizophrenia in the schizophrenia-enriched cluster and the individuals with no major psychiatric diagnosis, and 26 differentially expressed probes were identified in the patients with schizophrenia. Interestingly, 12 of the 26 identified genes were related to neurogenesis and cell-maturation/migration; these 12 included calbindin whose expression was reduced by >60% in the schizophrenia-enriched cluster. In a similar, independent study, Altar et al. conducted DG transcriptome analysis in human patients with schizophrenia [43] and indicated that calbindin was significantly downregulated in the DG of these patients. 

Patients with schizophrenia and those with bipolar disorder have also been examined for the molecular characteristics of the iDG phenotype. Postmortem immunohistochemical analysis of brain tissue revealed that patients with schizophrenia and those with bipolar disorder display significantly elevated calretinin expression in the DG, compared to both control subjects and patients having major depression. The increase of calretinin is strongly correlated with a history of psychosis. Moreover, compared to both control subjects and patients having major depression, patients with schizophrenia and those with bipolar disorder have significantly elevated ratio of calretinin to calbindin [7]. These facts demonstrate that an iDG-like condition may be also found in human patients with schizophrenia and/or bipolar disorder.

**Figure 6:**
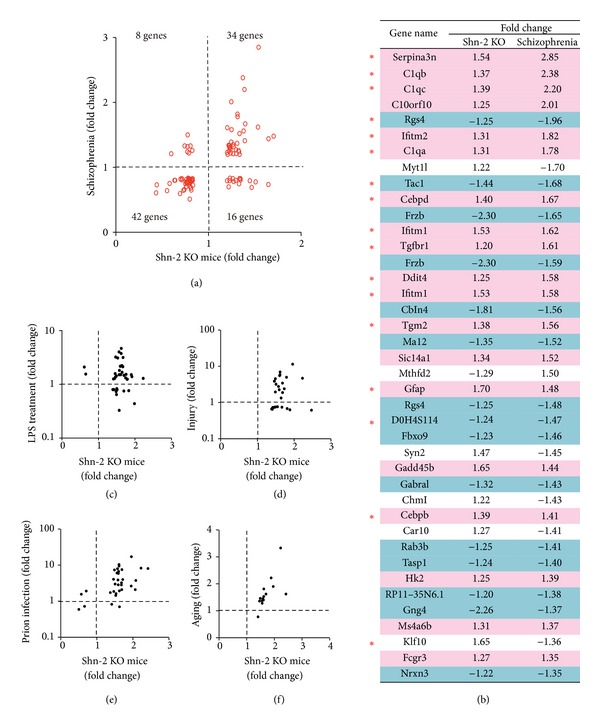
Comparison of gene expression profiles between Shn-2 KO mice and individuals with schizophrenia. (a) Scatter plot of gene expression fold change values in the medial prefrontal cortex (mPFC) of Shn-2 KO mice and Brodmann area (BA) 10 of postmortem schizophrenia brain. (b) Genes differentially expressed in both Shn-2 KO mice and in schizophrenia. Red indicates gene upregulation and blue indicates downregulation in both Shn-2 KO mice and in schizophrenia. The top 40 genes with respect to the fold change values are included. Asterisks indicate inflammation-related genes. (c)–(f) The hippocampal transcriptome pattern of Shn-2 KO mice was similar to the transcriptome data from lipopolysaccharide (LPS) treatment ((c), *P* = 5.6 × 10^−9^), injury ((d), *P* = 5.7 × 10^−24^), prion infection ((e), *P* = 1.0 × 10^−18^), and aging ((f), *P* = 1.4 × 10^−26^). Adapted from Takao et al. [2].

A mouse model of schizophrenia that expresses the iDG phenotype (Shn-2 KO mice) has been shown to display a series of brain phenotypes found in schizophrenia. These phenotypes include decreased gamma-aminobutyric acidergic (GABAergic) neuronal molecules (i.e., PV, glutamic acid decarboxylase isoform 67 (GAD67), *Gabra1*, etc.), reduction of oligodendrocyte markers (myelin basic protein and 2′,3′-cyclic-nucleotide 3′-phosphodiesterase (CNPase)), thinner cortex, and abnormal electroencephalographic findings (increased theta waves and decreased gamma waves). The gene expression pattern in Shn-2 KO mice was compared with that in patients with mental disorders obtained from publicly available array data using the bioinformatics tool, NextBio (Cupertino, CA, USA). NextBio is a repository of analyzed microarray datasets that allows an investigator to search results and the expression profiles of publicly available microarray datasets. This analysis determined that the highest degree of gene expression overlap was detected from Broadmann area 10 (BA10) in postmortem tissue from patients with schizophrenia and from control subjects, with 100 genes commonly altered in both Shn-2 KO mice and patients with schizophrenia [2]. The similarity was extraordinal (*P* = 9.5 × 10^−14^), which is unlikely to be obtained by chance. Furthermore, the expression levels of 76 of these 100 genes were altered in the same directions (Figures 6(a) and 6(b)). These findings support the idea that iDG or an equivalent phenomenon could also be present in human brain.

## 6. Maturation Abnormalities of Neurons other than DG Granule Cells in Humans with Psychiatric Disorders

Growing evidence from postmortem research implicates abnormal neurodevelopment in the pathogenesis of schizophrenia and other psychiatric disorders. In addition to detection of the iDG-like condition mentioned earlier, immaturity of neurons other than DG granule cells has also been shown in the brains of patients with psychiatric disorders. 

The maturation of GABA signaling is characterized by progressive switches in expression from GAD25 to GAD67 and from NKCC1 to KCC2 in the prefrontal cortex (PFC) and hippocampus of human brain. GAD25 is predominantly expressed in the fetus, whereas GAD67 is stably expressed across development [44]. GABA synthesis is increased concurrently with the switch from GAD25 to GAD67. The cation-chloride cotransporters, NKCC1 and KCC2, contribute to change of GABA from an excitatory to an inhibitory neurotransmitter in developing brain. NKCC1 expression predominates early in the developmental period and KCC2 expression rises as brain development progresses [44, 45]. In the hippocampus of patients with schizophrenia, GAD25/GAD67 and NKCC1/KCC2 ratios are increased and KCC2 expression is significantly decreased, indicating a potentially immature state in a certain type of GABAergic neurons [44]. 

Decreased levels of PV have been shown in brains of individuals with schizophrenia [46–49] and bipolar disorder [50]. Because PV is known to be a marker of mature FS inhibitory neurons, FS cells were hypothesized to be immature in these psychiatric disorders [51, 52]. In this context, Gandal et al. developed an FS cell maturation index. Using time-course gene expression data from developing FS cells that were positively correlated with PV expression levels, they showed a reduction of the index in cortices of patients with schizophrenia, bipolar disorder, and autism [52]. These results suggest that FS neurons are arrested in an immature-like state in the cortices of patients with these psychiatric disorders.

Furthermore, reduction of PNNs has been reported in the lateral nucleus of the amygdala and in layer II of the lateral entorhinal cortex of subjects with schizophrenia [38, 53]. PNNs are chondroitin sulfate proteoglycan-(CSPG-) containing neural extracellular matrices and are particularly enriched around PV-positive inhibitory neurons. Because numbers of PV-positive cells are normal within these regions in schizophrenia, the reduction of PNNs indicate downregulated CSPG levels, rather than loss of PNN-associated neurons [53, 54]. Disruption of PNN formation is thought to lead maturation abnormalities in selective neuronal populations, including those expressing PV [39, 40]. Since PNNs develop late in postnatal development and coincide with the maturation of PV-positive neurons [38, 40], the reduction of PNNs may imply that the PV-positive neurons remain at an immature-like state in the amygdala and entorhinal cortex of subjects with schizophrenia.

Taken together, these findings suggest that immaturity of neurons in adult brain can be seen, not only in the DG, but also in other brain regions in humans with psychiatric disorders. Further studies that focus on defining the maturation state of cells could identify maturation abnormalities of cells in additional brain regions.

## 7. Potential Mechanisms Underlying iDG

When attempting to fully understand the iDG phenotype, a logical next question is: what are the underlying mechanisms causing iDG? Both genetic and pharmacological manipulations have been shown to induce the iDG phenotype, and these factors could provide clues towards identifying the mechanisms behind this phenotype.

### 7.1. Inflammation

One clue into these mechanisms may lie in the normal functions of genes that, when altered, produce mouse models that express the iDG phenotype. For example, Shn-2 can be linked to the inflammatory cascade in that Shn-2 was originally identified as a nuclear factor-kappa B (NF-*κ*B) site-binding protein that tightly binds to the enhancers of major histocompatibility complex (MHC) genes in the MHC regions of chromosome 6 [55]. Recent genome-wide association studies have identified a number of single nucleotide polymorphisms (SNPs) in the MHC region associated with schizophrenia [56–60]. There are several MHC class I genes (*HLA-A*, *HLA-B*, etc.), each with similar promoter structures that have 2 NF-*κ*B binding motifs [61]. Upon inflammation, NF-*κ*B is known to bind to these enhancers and to initiate transcription of genes involved in immune functions. Shn-2 binds these motifs but does not substitute for the transcriptional function of NF-*κ*B [62]. Instead, Shn-2 competes with NF-*κ*B for binding to the motifs and inhibits NF-*κ*B activity. It has been reported that Shn-2 KO mice have constitutive NF-*κ*B activation and that T-cells of these mice show dramatic enhancement in differentiation towards T-helper Type 2 cells (Th2), a phenomenon called “Th2 slant,” [63] which is observed in patients with schizophrenia [64]. Thus, Shn-2 is an inhibitor of NF-*κ*B, a major mediator of inflammation. 

Due to the link between Shn-2 and inflammation, altered gene expression patterns were expected in Shn-2 KO mice. Using the bioinformatics tool, NextBio, publicly available datasets were analyzed to find the conditions where transcriptome patterns are similar to those found in the PFC of Shn-2 KO mice. Gene expression patterns in the PFC of Shn-2 KO mice were found to be similar to those seen in inherently inflammatory conditions (e.g., prion infection, malaria encephalopathy, spinal cord injury, brain injury, and kidney tissue treated with lipopolysaccharide) (Figures 6(c)–6(f)). However, this analysis highlighted that the scale of inflammation for Shn-2 KO mice is quite different from other inflammatory conditions (i.e., the inflammation in Shn-2 KO mice is much weaker in terms of the magnitude of the changes). Additionally, it was found that many of the genes with altered expression in both Shn-2 KO mice and patients with schizophrenia were inflammation-related genes (Figure 6(b)). Astrocytic activation is considered an indication of inflammation and GFAP, a reactive astrocyte marker, is increased not only in Shn-2 KO mice but also in SNAP-25 KI [3], FLX-treated [65], and pilocarpine-treated mice [33]. Therefore, the iDG phenotype can be associated with inflammation-like phenomena. 

### 7.2. Neuronal Hyperexcitation

In pilocarpine-induced epilepsy mouse models, a single episode of status epilepticus (SE) was not enough to induce the iDG phenotype. Chronic models of animal epilepsy develop spontaneous recurrent seizures (SRS) following SE over a period of months or years. In mice with pilocarpine-induced epilepsy, the markers of the iDG phenotype were identified in mice that developed SRS following SE, but not in mice that, after pilocarpine-induced SE, did not develop SRS [6]. These findings have 2 potential interpretations: (1) tonic/chronic hyperexcitation of hippocampal neurocircuits leading to SRS is needed to induce the iDG phenotype, or (2) the iDG phenotype itself is necessary for the development of SRS. However, a representative model of iDG (*α*-CaMKII HKO mice) has a more than 4-fold increase in sensitivity to pilocarpine for triggering seizures, but not developing SRS.

 Recently, it has been reported that SNAP-25 KI mice sometimes exhibit spontaneously occurring convulsive seizures after postnatal days 21–24 [66]. These seizures can be suppressed with chronic administration of valproic acid, an anticonvulsant, starting at postnatal day 16 [67]. These findings led to an investigation of whether iDG phenotypes and behavioral abnormalities could be rescued in mutant mice by suppression of recurrent epileptic seizures with valproic acid. Results showed that in the DG of SNAP-25 KI mice decreased expression of calbindin and increased expression of calretinin could be rescued with chronic administration of valproic acid. Valproic acid treatment also reduced the previously observed increase in the size of the DG and significantly improved working memory in these mice [3]. These results suggest that recurrent epileptic seizures underlie “dematuration” of DG granule cells, which induce the iDG phenotype.

Hyperexcitability of the DG granule cells is a feature shared among mice that express the iDG phenotype. The threshold current required to induce an action potential is reduced in *α*-CaMKII HKO, Shn-2 KO, SNAP-25 KI, and FLX-treated mice (Table 1). Furthermore, the increased sensitivity to pilocarpine (with respect to the seizure threshold) in *α*-CaMKII HKO mice supports the idea that hyperexcitation induces the iDG phenotype [6]. Previous studies have reported region-specific loss of GABAergic inhibition following SE/SRS, especially from the CA1, CA3, and hilar regions in the hippocampus. Notable molecular/morphological deficits observed in iDG mouse models include a reduction of the GABAergic system in the hippocampus (e.g., a reduction of PV-positive interneurons and decrease of GAD67 expression, Takao et al. [2]). Although it is probably too simplistic to conclude (or even to speculate) that a simple loss of GABAergic cells and/or function from the hippocampus may contribute to induction of the iDG phenotype, these data suggest that sustained hippocampal neuronal hyperexcitation, caused by loss of GABAergic inhibition, may be a common driving force for initiating a cascade of molecular events that lead to development of the iDG phenotype. Collectively, these findings support the idea that neuronal hyperexcitation leads to “dematuration” of mature granule cells in the DG, thereby contributing to iDG phenotypes.

It should also be noted that iDG phenotypes are usually associated with mild chronic inflammation. Shn2-KO, SNAP-25 KI, pilocarpine-treated, FLX-treated, and paroxetine-treated mice commonly demonstrate upregulated expression of complement and MHC genes and GFAP, suggesting that neuronal hyperexcitation is a source of inflammatory-like processes in the brains of these mice. In contrast, recent research has highlighted the involvement of the immune system in the development of epilepsy. For instance, inflammatory cytokines are increased in the central nervous system (CNS) and plasma of epilepsy model animals and of patients with epilepsy [68, 69]. Moreover, inflammation in the CNS is associated with damage to the blood-brain barrier (potentially leading to leakage) that is known to enhance neuronal excitability and has been implicated in epileptogenesis [70–72]. In this context, Fabene et al. demonstrated that pilocarpine-induced SE enhances leukocytic inflammatory changes in the CNS vasculature, consequently generating epileptic activity [73]. Based on these data, a possible positive feedback mechanism between neuronal hyperexcitation and inflammatory conditions could subsequently induce iDG phenotypes.

More recently, it has been reported that knockdown of *Disc1* (disrupted in schizophrenia 1), specifically in adult-born DG neurons, results in enhanced excitability [74]. The *Disc1* gene was originally discovered in a Scottish family with a high incidence of psychiatric disorders, including schizophrenia and bipolar disorder [75]. *Disc1* knockdown also caused pronounced cognitive and affective deficits, which could be reversed when affected DG neurons were inactivated [74]. These findings are consistent with the hypothesis that hyperexcitation of DG neurons may underlie behavioral abnormalities related to schizophrenia and other neuropsychiatric disorders. 

### 7.3. Other Clues

The iDG phenotype is associated with several other cellular/molecular alterations, and these changes may serve as clues to the mechanisms underlying the phenotype. The GluR1 subunit of *α*-amino-3-hydroxy-5-methyl-4-isoxazole propionic acid-type glutamate receptor is dramatically reduced in most, but not all, mice that express the iDG phenotype. For example, it has been reported that GluR1 expression is reduced in the DG of *α*-CaMKII HKO [32] and Shn-2 KO [2]. Interestingly, GluR1 is expressed primarily in mature granule cells and can be used as a marker for maturation of granule cells [32]. Physiologically, Ca^2+^-permeability mediated by GluR1 and/or GluR3 subunits is strongly associated with granule cell maturation [76–78]. The GluR1 reduction demonstrated in these mutants may be consistent with the idea that DG granule cells are immature in these mice.

Arc and c-Fos are immediate-early genes (IEGs) that are commonly used as markers for the maturity of in vivo activity-dependent responsiveness of granule cells, whose expression can be stimulus-induced in 3- to 5-week-old cells [79, 80]. As mentioned previously, Arc promoter activity was dramatically reduced in the DG of *α*-CaMKII mice after performing working memory tasks [13]. Likewise, Arc induction after exposure to a novel environment or receiving electrical foot shocks was reduced in the DG of Shn-2 KO [2] and SNAP-25 KI mice [3]. In FLX-treated mice, expression of c-Fos was reduced after receiving foot shocks [5]. Since activity-dependent processes have been implicated in the maturation of adult neural progenitors, if reduction of IEG expression (or, conversely, promotion of expression) represents a state of low-responsiveness to behavioral stimulations in the granule cells of mice expressing the iDG phenotype, activity-dependent maturational mechanisms are also likely impaired in these cells. However, granule cells are more excitable in *α*-CaMKII HKO [1], Shn-2 KO [2], SNAP-25 KI [3], and FLX-treated mice [5]. Therefore, suppressed IEG induction in mice expressing the iDG phenotype might be the result of decreased activity and/or impairments in activity-dependent gene regulation in the DG.

Adult neurogenesis in the DG was assessed using incorporation of 5-bromo-2′-deoxyuridine (BrdU) and was elevated in *α*-CaMKII HKO [1], FLX-treated [25], and pilocarpine-treated mice. In the pilocarpine model of epilepsy, BrdU incorporation was increased in the acute phase after SE [33], as well as in the period of SRS [35]. However, despite the occurrence of epileptic seizures in SNAP-25 KI mice, reduced adult neurogenesis was observed in these mutants [3]. Further studies are needed to fully assess whether altered adult neurogenesis is involved in formation of the iDG phenotype. Electrophysiologically, an increase in granule cell excitability and a reduction of frequency facilitation at mossy fibre-CA3 synapses were found in *α*-CaMKII HKO, Shn-2 KO, SNAP-25 KI, and FLX-treated mice. These shared molecular and physiological alternations in mice with the iDG phenotype may provide important clues to investigate the mechanisms underlying iDG. 

Table 1 summarizes the behavioral, electrophysiological, and molecular patterns in different mice with the iDG phenotype. Some endophenotypes are perfectly shared, but others are not. There appear to be subgroups of the iDG phenotype, which may potentially correspond to different types of psychiatric disorders. Furthermore, there are likely some shared and some unshared mechanisms between the different groups of mice with the iDG phenotype.

## 8. Face and Construct Validity of iDG Mouse Models as Models of Psychiatric Disorders

Mice with the iDG phenotype possess face validity as animal models of schizophrenia at both the behavioral and molecular levels. In addition, various phenomena accompanied by the iDG phenotype are in good agreement with most of the major hypotheses of the disorder, supporting the construct validity of these models.

### 8.1. Face Validity

As mentioned earlier, mutant mice with the iDG phenotype (CN KO, *α*-CaMKII HKO, Shn-2 KO, and SNAP-25 KI mice) have been identified as potential models of schizophrenia and bipolar disorder, originally by behavioral screening designed to assess the face validity of this model. These mutant mice strains commonly exhibited severe working memory deficits, decreased PPI, impaired social behaviors, and hyperactivity. Impairments in working memory [81, 82], PPI [83], and social withdrawal [84] are prominent features of schizophrenia symptomatology. Hyperactivity is characteristic of rodent models of schizophrenia [85] and could correspond to the psychomotor agitation often present in patients with schizophrenia. Furthermore, FLX-treated and pilocarpine-treated mice express iDG and behavioral phenotypes shown by mutant iDG mice (Table 1). Therefore, when evaluated at a behavioral level, mice expressing the iDG phenotype display a strikingly conserved behavioral phenotype. 

Among these models, Shn-2 KO mice showed outstanding face validity as an animal model of schizophrenia. In addition, with respect to molecular profiles, Shn-2 KO mice shared altered molecular expression patterns with postmortem brain tissue from patients with schizophrenia. Significant similarities in expression direction and magnitude were detected when the gene expression pattern in Shn-2 KO medial PFC (mPFC) was compared with the pattern in schizophrenic frontal cortex (Brodmann area 10, BA10) examined postmortem [86]; 100 altered genes were detected in both Shn-2 KO mice and patients with schizophrenia. Of the 100 altered genes, 76 showed the same directional change in expression and, of the 76 genes, 42 were downregulated and 34 were upregulated [2]. A large number of genes previously implicated in schizophrenia or bipolar disorder were included in these groups. For example, 89 of the 100 similarly expressed genes have been identified as related to these disorders. Shn-2 KO mice also displayed decreased levels of PV in the frontal cortex, which has been widely observed in schizophrenia [46–49]. Fast-spiking PV-positive interneurons have been shown to be immature in the cortices of patients with schizophrenia, bipolar disorder, and autism [52]. This suggests that immaturity of this type of neuron in the cortex may underlie some of the cognitive deficits seen in these disorders. Shn-2 KO mice also exhibit decreased expression of PV and GAD67 in the hippocampus, which has been observed in the brains of patients with schizophrenia examined postmortem [87–89]. Furthermore, Shn-2 KO mice had significantly thinner cortex and reduced cell density in the prelimbic and primary visual cortices, consistent with observations in human patients with schizophrenia [90]. 

In addition to anatomical abnormalities, the cortex of Shn-2 KO mice exhibits physiological alternations. Cortical EEG analysis in mutant mice showed an increase in slow waves and a decrease in fast waves, both of which are observed in patients with schizophrenia [91–93]. Although present studies have evaluated the molecular and physiological validity using Shn-2 KO mice in detail, future studies are needed to address whether other mouse strains that express the iDG phenotype similarly fulfill the criteria for face validity. 

### 8.2. Hyperdopaminergic/Hypoglutamatergic Hypotheses

The longstanding “dopamine hypothesis of schizophrenia” is based primarily on 2 facts: (1) all antipsychotics currently available to treat the positive symptoms of schizophrenia possess dopamine D2 receptor blocking activity and (2) hyperactive dopamine release in subcortical limbic brain regions, especially in the nucleus accumbens/striatum, has been consistently observed in patients with schizophrenia [94, 95]. Dopamine D2 blockers are widely used for treating bipolar mania and hyperactivity of the dopaminergic system during manic phases has also been proposed [96]. A hyperdopaminergic state in subcortical regions is believed to be associated with psychosis, a core constituent of positive symptoms of schizophrenia and a phenotype often observed in manic phase of the bipolar disorder, and a symptom that can be ameliorated using treatment with D2 blockers. 

Intriguingly, *α*-CaMKII HKO mice have been proposed as a model of schizophrenia by Novak and Seeman [97], based on their finding that D2High receptors were elevated in the striatum (representing hyperdopaminergic state analogous to schizophrenia) of this mutant strain. Increasing evidence also supports that mice with iDG phenotypes are in a hyperdopaminergic state. For example, administration of haloperidol, a typical antipsychotic, significantly improved PPI impairment in Shn-2 KO mice [2]. Currently available antipsychotics (including haloperidol) work primarily by antagonizing dopamine D2 receptors and raising intracellular cAMP levels. Therefore, intracellular stimulation of cAMP levels is thought to have effects similar to treatment with antipsychotic medication [98, 99]. Rolipram is a cAMP-specific phosphodiesterase inhibitor that elevates intracellular cAMP levels. Chronic treatment with rolipram (in combination with ibuprofen) rescued a subset of mice expressing the iDG phenotype and improved working memory and nest building in Shn-2 KO mice. These findings suggest that lower intracellular cAMP levels are involved in a subset of behavioral abnormalities and iDG phenotypes. 

Interestingly, dopamine D1 signaling is also upregulated in the DG of *α*-CaMKII HKO and Shn-2 KO mice, when assessed using receptor binding assay and receptor agonist sensitivity, respectively. Increased expression of *Drd1a* mRNA also suggests elevated D1 signaling in *α*-CaMKII [1], SNAP-25 KI [3], FLX-treated [65], and pilocarpine-treated mice [6]. Upregulated D1 signaling might serve as a compensatory mechanism to normalize intracellular cAMP levels in these mice. These results suggest the presence of a hyperdopaminergic state in the brains of mice with the iDG phenotype. 

This hyperdopaminergic state is thought to be a consequence of NMDA receptor hypofunction. This assertion is supported by findings showing that acute application of an NMDA receptor antagonist stimulates dopamine release in both animal models and humans. CaMKII is a major downstream molecule of the NMDA receptor and plays an important role in long-term potentiation and long-term depression [100]. Since deficiency of *α*-CaMKII causes schizophrenia-related behavioral and iDG phenotypes, NMDA receptor hypofunction might be involved in the formation of the iDG phenotype. In addition, in *α*-CaMKII HKO mice, NMDA receptor- binding was also downregulated in the hippocampus, especially in the DG [1], supporting the idea of NMDA receptor hypofunction in these mutant mice. Additional evidence for this hypothesis includes the fact that MK-801, an NMDA receptor antagonist that causes schizophrenia-like psychosis in humans, produced significantly higher levels of drug-stimulated motor activation in CN KO [10] and Shn-2 KO mice [2]. Taken together, these findings suggest that hypoglutamatergic/hyperdopaminergic function is involved in the iDG phenotype.

### 8.3. Neurodevelopmental/Neurogenesis Hypothesis

The neurodevelopmental hypothesis is one of the major theories regarding the pathogenesis of schizophrenia [101–103]. This hypothesis is supported by several lines of evidence, including increased frequency of obstetric complications in patients with schizophrenia, the presence of neurological, cognitive, and behavioral dysfunction long before illness onset, the absence of postmortem evidence of neurodegeneration, and the induction of schizophrenia-like phenomena by neonatal hippocampal lesions in model animals [101, 103–105]. These findings are consistent with a neurodevelopmental model in which the etiology of schizophrenia may involve pathologic processes caused by both genetic and environmental factors that begin before adolescence when the brain approaches its adult anatomical state.

The iDG phenotype literally reflects a neurodevelopmental problem in which DG granule cells arrest in a pseudoimmature state. Takao et al. revealed that iDG phenotypes emerge at 2–4 weeks of age in Shn-2 KO mice [2]. In 2-week-old mice, there were no significant differences in the expression of calbindin and calretinin in the DG of Shn-2 KO and wild-type mice. However, when mice were evaluated at 1 month of age, calbindin expression was decreased and calretinin was increased in the DG of Shn-2 KO mice, indicating the postnatal (possibly adolescent) emergence of an iDG phenotype.

Specifically, the number of calbindin-positive cells was decreased at age of 4 weeks, compared to age of 2 weeks in Shn-2 KO mice, whereas, in wild-type mice, these cells increased throughout development. Even in adult wild-type mice, “dematuration” of mature granule cells and schizophrenia-related behavioral phenotypes, could be induced by chronic FLX treatment [5] or with pilocarpine-induced SRS [6]. These findings suggest that genetic and/or environmental factors could induce the maturational abnormalities of DG granule cells during the postnatal developmental period (and during adulthood).

Elevated neurogenesis has been defined as a core feature of the pathology associated with the iDG phenotype, with few exceptions (see [3]). One study supports a linkage between schizophrenia and decreased hippocampal neurogenesis, as tissues from human patients with schizophrenia had fewer proliferating Ki-67 cells than tissues from control subjects [112]. However, that study excluded several patients with neurogenesis levels greater than 5-fold baseline levels, which may represent a subset of patients with schizophrenia/bipolar disorder that display hyperactive neurogenesis. 

### 8.4. Inflammation Hypothesis

The well-established role of inflammation in the etiology of schizophrenia is often referred to as the inflammation hypothesis [64, 113–115]. The link between prenatal infection and schizophrenia was first identified in an epidemiological study demonstrating increased schizophrenia risk in the offspring of women exposed to influenza during pregnancy [64]. Several other infectious factors have also been implicated in the pathogenesis of schizophrenia [64], suggesting that the disease may result from maternal immune response to infection. To this end, prenatal treatment with polyinosinic:polycytidylic acid (poly I:C), viral infection, and LPS treatment are used as rodent models of schizophrenia [114, 116–119]. As mentioned previously, Shn-2 KO mice were shown to exhibit mild chronic inflammation of the brain, as evidenced by increased inflammation markers (including complement and MHC class I genes, GFAP, and NADH/NADPH oxidase p22-phox) and genome-wide gene expression patterns similar to various inflammatory conditions [2]. Similarly, SNAP-25 KI mice, which develop epileptic seizures, showed increased expression of complement and MHC class I genes, and *GFAP* in the hippocampus [3]. In FLX-treated [65], paroxetine-treated [31], and pilocarpine-treated animals [34], gene chip data reveals hippocampal upregulation of complement and MHC genes, and *GFAP*. Therefore, the changes in inflammation-related molecules in these mice with iDG are consistent with inflammatory features found in the brains of patients with schizophrenia and epilepsy.

### 8.5. Collection of Genetic Association Study Results

Schizophrenia is highly heritable and multiple genetic and environmental factors are probably be involved [120]. Mice with iDG phenotype exhibit significantly altered expression of genes that have been reported to associate with schizophrenia. 

For example, mutated genes that cause iDG phenotype have been implicated in schizophrenia by association studies. Based on the observation that CN KO mice display a spectrum of behavioral abnormalities strikingly similar to those observed in patients with schizophrenia [21], Gerber et al. examined whether CN dysfunction is involved in the etiology of schizophrenia [9]. By conducting transmission disequilibrium studies in a large sample of affected families, they reported evidence supporting an association between the *PPP3CC* gene encoding the CNA-gamma catalytic subunit and schizophrenia in populations from the United States and South Africa. The allelic and haplotypic associations of *PPP3CC* with schizophrenia were later confirmed in populations from Japan [20]. Thus, CN KO mice have good construct and face validity as a model for schizophrenia. 

Moreover, recent genome-wide association studies have identified a number of SNPs in the MHC region associated with schizophrenia [56–59]. Many of these SNPs are located in or near NF-*κ*B binding sites that may be associated with susceptibility to schizophrenia [2]. Some of the genes surrounding these SNPs are dysregulated in the brains of patients with schizophrenia examined postmortem and/or the PFC of Shn-2 KO mice. As Shn-2 is a MHC enhancer-binding protein that binds to the NF-*κ*B binding motif, abnormal transcription of these genes may be induced by dysregulated NF-*κ*B signaling pathways, independent of any deficiency or mutation in Shn-2 itself. 

In addition, recent human genetic studies have discovered associations between SNAP-25 and various psychiatric and neurological disorders, including schizophrenia [14, 15], ADHD [16, 17], and epilepsy [18, 19]. Translational convergent functional genomic study demonstrates that SNAP-25 is one of the top 42 candidate genes for schizophrenia [121]. Therefore, SNAP-25 KI mice also have strong construct validity for schizophrenia. 

Many of the genes with decreased expression in both schizophrenic BA10 and Shn-2 KO mPFC have been reported to be associated with schizophrenia. Chowdari et al. reported that several SNPs in *RGS4*, regulator of G-protein signaling-4, are associated with schizophrenia [122]. It has been previously shown that *RGS4* expression is decreased across 3 cortical areas (including the PFC) of subjects with schizophrenia [123]. Consistent with these observations, *RGS4* mRNA expression was decreased in the mPFC of Shn-2 KO mice [2]. Additionally, significant associations were detected between samples from patients with schizophrenia and SNPs in* Cbln4 *[57], *Gabra1 *[124], and *Nrxn3 *[57]. These genes are involved in synaptic transmission or synaptic plasticity, and expression of these genes is commonly decreased in both schizophrenic BA10 and Shn-2 KO mPFC. Thus, decreased expression of genes associated with schizophrenia could play a significant role in developing schizophrenic conditions in the PFC of iDG mice, especially of Shn-2 KO mice. 

Genes related to neuronal excitation and inflammatory responses have been reported to be associated with schizophrenia [125]. As previously mentioned, these conditions are closely related to induction of the iDG phenotype. A meta-analysis of clinical data showed an association between SNPs in *KCNH2* (a human Ether-à-go-go-family potassium channel) and schizophrenia [126]. The *KCNH2*-3.1 isoform modulates neuronal firing, and its expression is increased in the hippocampi from individuals with schizophrenia, suggesting increased neuronal excitability in the schizophrenic brain [126]. 

Regarding inflammatory or immune response-related genes, as previously mentioned, a number of SNPs in the MHC region are associated with schizophrenia. In addition, many inflammatory response-related genes such as *C1QA*, *C1QB *[127], *TAC1 *[128, 129], *TGFBR1 *[129], and *TGM2 *[130] have been associated with schizophrenia. *GFAP* is also known to have a significant association with schizophrenia [15]. These mRNA expression changes were in the same direction as changes seen in schizophrenic BA10 and Shn-2 KO mPFC [2]. Thus, it has been established that altered expression of genes related to neuronal excitation and inflammatory conditions could be involved in the brains of patients with schizophrenia as well as in mice with iDG phenotype. 

Taken together, these findings indicate that mice with the iDG phenotype have high construct validity as animal models of schizophrenia, with many of the genes showing a significant association with schizophrenia. 

### 8.6. Other Factors

Some other factors, such as dysfunction of oligodendrocytes or mitochondria, have been implicated in psychiatric disorders. Several potential deficits were observed in key oligodendrocyte markers, including decreases in CNPase and myelin basic protein (MBP), which is a major constituent of the myelin sheath of oligodendrocytes and Schwann cells. Decreased CNPase protein levels have been reported in schizophrenia [131]. Similarly, *MBP* mRNA levels were decreased in the DG as well as the mPFC of Shn-2 KO mice, and proteome analysis reveled that many mitochondria-related proteins (e.g., dihydrolipoamide *S*-acetyltransferase (DLAT), ubiquinol-cytochrome *c* reductase core protein (UQCRC), and NADH dehydrogenase [ubiquinone] Fe-S protein (NDUFS)) in the DG are affected in Shn-2 KO mice [2, 132]. These findings suggest that changes in the expression of molecules related to oligodendrocytes or mitochondria in the DG of Shn-2 KO mice would be consistent with the oligodendrocyte and mitochondria hypotheses of schizophrenia, respectively.

**Figure 7:**
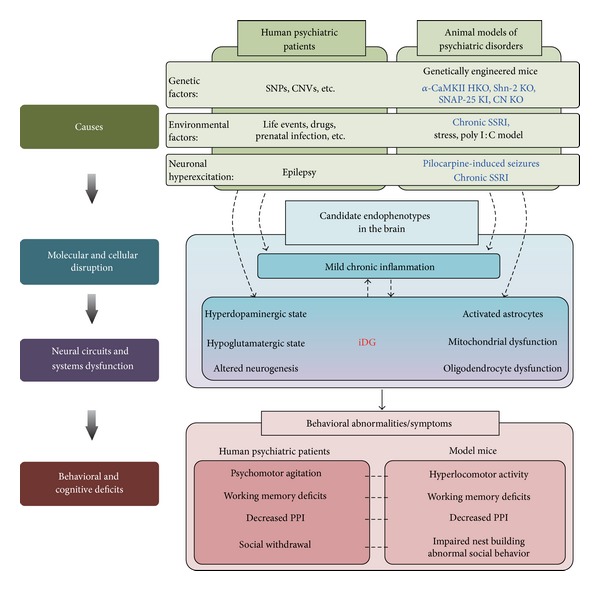
A schematic representation of the model of involvement of the iDG phenotype in psychiatric disorders. In psychiatric disorders, such as schizophrenia, bipolar disorder, and depression, multiple genetic and environmental factors could induce mild chronic inflammation. This could subsequently result in multiple endophenotypes in the brain, including iDG. Inflammation could induce alterations in neurogenesis [106, 107], mitochondrial dysfunction [108, 109], and oligodendrocyte dysfunction [110, 111]. Involvement of inflammation in induction of a hypoglutamatergic state or hyperdopaminergic state remains unclear. These possible endophenotypes may affect each other, which may, in turn, cause behavioral abnormalities in psychiatric patients. There may not be a single “principal” or “core” mechanism that underlies the behavioral symptom of psychiatric disorders. Indeed, a shared or preserved set of multiple endophenotypes, as a whole, may be the principal mechanisms of these disorders.

Figure 7 shows a schematic representation of the model detailing how the iDG phenotype may be involved in schizophrenia, bipolar disorder, and other psychiatric disorders. This proposed model is based on the findings that mice with the iDG phenotype possess face and construct validity as animal models of these disorders. In these disorders, multiple genetic and environmental factors could induce mild chronic inflammation, and this could subsequently result in multiple endophenotypes (including iDG) in the brain, that may, as a whole, cause behavioral abnormalities in psychiatric patients.

## 9. A Possible Role of iDG in Behavioral Abnormalities Relevant to Neuropsychiatric Disorders 

### 9.1. iDG and Working Memory

Impairment in working memory is one of the core behavioral abnormalities in mice with the iDG phenotype. CN KO [21], *α*-CaMKII HKO [1, 12], Shn-2KO [2], SNAP-25 KI mice [3], and pilocarpine-treated animals [30] have been reported to display severe working memory deficits in the spatial working memory version of the eight-arm radial maze and/or in the T-maze forced-alteration task. 

The hippocampal structure has been shown to be critically involved in working memory function [133, 134]. The DG is a key input node for the hippocampal structure. Perforant path fibers originating in the entorhinal cortex provide a major source of highly processed sensory information to DG granule cells. When rats with lesions in the DG were tested on working memory version of the eight-arm radial maze, they showed impairment in their ability to perform this version of the task [135–137]. Considering the evidence supporting involvement of the DG in working memory function, it is likely that the iDG phenotype has a negative impact on working memory function. However, the possibility that deficits in regions other than the DG cause impairments in working memory in the mice with the iDG phenotype cannot be excluded.

In working memory tasks, animals must rapidly establish and maintain a memory of visited areas based on single within-trial exposures and must suppress interference by memories obtained during previous trials. Deficits in either function could cause impairments in working memory tasks. Mice with the iDG phenotype commonly display a reduction of frequency facilitation at mossy fibre-CA3 synapses [1–3, 5]. Frequency facilitation is a potential mechanism for the involvement of the CA3 region in the processing of working memory, especially in quickly encoding novel information into the hippocampal memory system [138]. Therefore, the deficit might contribute to impairments in establishment and maintenance of memory in mice with the iDG phenotype. In addition, mice with the iDG phenotype might have deficits in processes that reduce memory interference. Interference between already remembered information and to-be-remembered information can have a profound effect on mnemonic accuracy [139]. DG granule cells have been proposed to play important roles in temporal pattern separation, in which an initial event should be separated from a later event [140, 141]. If this hypothesis proves true, mice with the iDG phenotype might have deficits in suppressing memory interference, leading to impaired working memory. 

### 9.2. iDG, Pattern Separation, and Psychosis

Among hippocampal subfields, computational models have highlighted the essential role of the DG for disambiguating similar events. This so-called pattern separation refers to the computational process for making representations for similar input patterns more orthogonal for better discrimination [142–145]. Experimental efforts have tested these computational hypotheses using hippocampal region-selective lesions [146–149], *in vivo* electrophysiological recording [150], and genetic manipulation [151]. More recently, investigation of adult-born DG neurons in memory formation has focused on specific function-pattern integration and pattern separation [152, 153]. Marín-Burgin et al. showed enhanced excitation/inhibition balance and low input specificity in immature granule cells [153]. These electrophysiological features of immature granule cells have been proposed to be crucial for pattern integration [153]. For example, mature granule cells are presumably very selective in their responses, which may contribute to pattern separation by making the stimulus inputs independent of one another. In contrast, the physiological properties of immature granule cells probably make them less selective, which may result in firing in response to multiple events [152, 153]. 

As mentioned earlier, granule cells in the pseudoimmature state are hyperexcitable in *α*-CaMKII HKO [1], Shn-2 KO [2], SNAP-25 KI [3], and FLX-treated mice [5]. Given that the physiological properties of pseudoimmature granule cells are comparable to those of immature granule cells in normal development, it is tempting to hypothesize that, due to a lack of appropriate memory separation, the iDG phenotype causes excessive integration and association of memories to actual and imaginary events. 

However, Nakashiba et al. have reported that pattern separation requires adult-born immature granule cells, not mature granule cells [154]. The role of immature and pseudoimmature granule cells on pattern separation/integration remains controversial. Therefore, future studies are needed to examine input selectivity of pseudoimmature granule cells in mice with the iDG phenotype and to address whether they exhibit deficits in pattern separation. 

An iDG signature, increased calretinin immunoreactivity, has been identified from patients with schizophrenia and bipolar disorder. The increase in the calretinin signal was positively correlated with a history of psychosis in these patients [7]. Similarly, in iDG mouse models, hyperlocomotor activity, generally thought to be linked to a hyperdopaminergic/psychosis state, has been consistently observed [1–3, 10]. Indeed, *α*-CaMKII HKO mice show hyperlocomotion and simultaneously display hypersensitivity to amphetamine-induced hyperlocomotion (Kogan & Matsumoto, unpublished observation), a well-established behavioral trait that is believed to reflect a hyperdopaminergic/psychosis state in patients with schizophrenia/bipolar disorder. Based on this evidence, iDG may represent a pathophysiological deficit shared among patients with psychosis, a deficit presumably linked to a hyperdopaminergic state. Interestingly, iDG signatures have also been identified in pilocarpine-induced epilepsy mouse models [6]. Epidemiological evidence indicates that psychosis is often comorbid with epilepsy and vice-versa [155]. Thus, these recent findings suggest that a common hyperdopaminergic state may further strengthen the link between epilepsy and psychosis [156]. 

How iDG contributes to psychosis and a hyperdopaminergic state remains an open question. While not fully answered, iDG may fit as a potential player in the disturbed neurocircuits, based on recent studies that have proposed that hippocampal neurocircuits influence dopaminergic neuronal tones that underlie psychotic and schizophrenic conditions [157, 158]. 

The consequences of iDG (specifically, excess immature/less-mature granule cells) on the function of the hippocampus also remain open questions. It has recently been proposed that immature granule cells in the DG may inhibit or destabilize activity of the entire DG via activation of inhibitory GABAergic input from hilar interneurons [159]. Because immature granule cells preferentially innervate hilar interneurons and these interneurons innervate all DG granule cells, excess immature neurons may consequently lead to gross inhibition of the DG. Although immature granule cells in iDG models are hyperexcitable compared to mature granule cells [1–3, 5], their hyperexcitability may cause further gross inhibition of DG by the proposed feedback mechanism from hilar interneurons. A synaptic competition for perforant path connections between older, more-mature granule cells and newly generated immature cells has also been proposed as a potential cause of the gross inhibition of the DG by immature granule cells [159]. In iDG mouse models, mature granule cells are dramatically reduced. This reduction of mature neurons could be easily connected to dysfunction of the DG, especially after considering the reduced arborization of DG granule cells in iDG models [1]. 

In iDG models, the DG also does not respond to stimulus (e.g., lack of c-Fos expression in DG following electric foot shock in *α*-CaMKII HKO mice [1] and dramatically reduced Arc induction in a novel environment in Shn-2 KO mice [2]). It currently remains unclear whether DG activity is actually decreased. Furthermore, it remains unclear if decreased DG activity leads to less glutamatergic input to CA3 from the DG through mossy fibers. Although mossy fibers from the DG to CA3 are glutamatergic and come into direct contact with CA3 pyramidal neurons, many mossy fiber synapses are connected to interneurons in CA3 that provide GABAergic inhibitory input to CA3 pyramidal cells. As a consequence, DG and mossy fiber activation causes a net inhibition within the CA3 network and presumably activates only a specific subset of CA3 pyramidal neurons [160, 161]. From this vantage point, net inhibition of the DG by immature granule cells may cause a tonic net activation of CA3. Induction of Arc transcription after foot shock was greatly suppressed in almost every region of the Shn-2 KO brain. Despite this near-ubiquitous reduction of Arc, comparable Arc induction was observed in the CA1 and CA3 regions of the hippocampus in Shn-2 KO and control mice. The relatively higher Arc induction in these regions suggests that CA areas in these mutant mice may be more active than other brain regions.

This functional failure in the hippocampal formation *per se* is proposed by Tamminga et al. [158] as an underlying mechanism of psychosis, in which hypoactive DG (weakened pattern separation) and hyperactive CA3 (strengthened pattern completion) confer psychosis (cognitive “mistakes”) in patients with schizophrenia and other psychiatric conditions. Since an iDG signature was identified in patients with schizophrenia and bipolar disorder and due to its strong association with psychosis, it is conceivable that iDG is one of the potential mechanisms causing dysfunction of the DG and subsequent tonic activation of CA3-CA1 in patients with psychiatric conditions.

As discussed earlier, a subcortical hyperdopaminergic state in patients is also believed to play a pivotal role in the development of psychosis and is the only biochemical finding supported by the mechanism-of-action of currently available drugs. A hypothesis has been proposed by Grace et al. [157, 162] suggesting that overdrive of the subcortical dopamine system is caused by hyperactivation of the ventral hippocampus. Tonic activation of ventral CA3-CA1 induced by the iDG phenotype theoretically leads to activation of the ventral subiculum (vSub) through direct glutamatergic transmission. Glutamatergic afferents to the nucleus accumbens (NAc) from the vSub then activate GABAergic projections from the NAc to the ventral pallidum, suppressing pallidal GABAergic efferent neurons. Because dopaminergic neurons in the ventral tegmental area (VTA) that project to the subcortical limbic region (including the NAc) are governed by GABAergic inhibitory projections from the ventral pallidum, activation of the NAc by the vSub suppresses tonic inhibitory GABAergic inputs from the ventral pallidum and consequently leads to tonic activation of VTA dopamine neurons. 

## 10. Conclusion

In the course of large-scale behavioral screening of mouse models of neuropsychiatric disorders, a number of strains of mice displayed a series of schizophrenia-related behavioral abnormalities, including increased locomotor activity, severe working memory deficits, and decreased social interaction. Among these models, *α*-CaMKII HKO, Shn-2 KO, SNAP-25 KI, and CN KO mice all possessed an iDG phenotype. Subsequently, it was shown that chronic FLX treatment or pilocarpine-induced SRS reverses neuronal maturation, resulting in the iDG phenotype in wild-type mice. Importantly, iDG-like phenomena have been observed in the brains of patients with schizophrenia and bipolar disorder when examined postmortem. Based on these observations, iDG is proposed as a potential endophenotype shared by certain types of neuropsychiatric disorders.

Due to limitations of the current psychiatric diagnostic methods, schizophrenia, bipolar disorder, and other related psychiatric disorders are considered biologically heterogeneous populations. Therefore, an endophenotype-based analysis would be preferable for establishing biological characteristics for the classification of psychiatric disorders, rather than an analysis based on current diagnostic methods [163]. In this context, the current findings suggest that the iDG phenotype could be used as a potential brain endophenotype shared by neuropsychiatric disorders (including schizophrenia, bipolar disorders, and epilepsy) and could be useful for phenotype-based classification of these disorders. Furthermore, at least partial rescue of iDG phenotypes and some behavioral abnormalities was possible in Shn-2 KO and SNAP-25 KI mice with treatment based on possible mechanisms underlying the iDG phenotype. Further investigation of iDG as an important factor in the precipitation of, as well as recovery from, episodes of schizophrenia and other psychiatric disorders would facilitate the study of these disorders.

## Figures and Tables

**Table 1:** Behavioral, electrophysiological, and molecular phenotypes in mice with iDG.

		*α*-CaMKII HKO [1]	Shn-2 KO [2]	SNAP-25 KI [3]	CN KO [4, 10, 21]	FLX treatment [5]	Pilocarpine treatment [6]
Behavioral phenotypes	Locomotor activity	↑	↑	↑	↑	↑↓ [29]	↑
	Working memory	↓ [1–12]	↓	↓	↓		↓ [6, 30]
	Social interaction	Mutants kill cagemates	↓	↓	↓		↓
	PPI	—	↓	↓	↓	—	

Electrophysiological phenotypes	*Mossy fibre-CA3 synapse *						
	Basal transmission	↑	↑	—		—	
	Frequency facilitation	↓	↓	↓		↓	—
	*Granule cell soma *						
	Passive						
	Input resistance	↑	↑	—		—	—
	Active						
	Excitability	↑	↑	↑		↑	↑
	Spike number	↓	↓	↑		↑	↑

Molecular phenotypes	*Markers of immature granule cells *						
	Dopamine D1 receptor (Drd1a)	↑	—	↑	↑	↑ [31]	↑
	Calretinin	↑	↑	↑	↑	↑	↑
	*Markers of mature granule cells *						
	Tryptophan 2,3-dioxygenase (Tdo2)	↓	↓	↓	↓	↓	↓
	Desmoplakin (Dsp)	↓	↓	↓	↓	↓	↓
	Calbindin	↓	↓	↓	↓	↓	↓
	GluR1	↓ [32]	↓		↓	—	
	*Immediate early genes induction *						
	c-Fos or Arc-dVenus	↓ [1, 13]	↓	↓	↓	↓	
	*Inflammation-related molecules *						
	Glial fibrillary acidic protein (GFAP)	—	↑	↑	↑	↑ [31]	↑ [33]
	Complement components	—	↑	↑		↑ [31]	↑ [34]
	MHC genes	—	↑	↑		↑ [31]	↑ [34]
	*Adult neurogenesis *						
	BrdU incorporation	↑		↓	↑	↑ [25]	↑ [33, 35]

↑: Increased or upregulated.

↓: Decreased or downregulated.

—: Nonsignificant.

## References

[1] Yamasaki N, Maekawa M, Kobayashi K (2008). Alpha-CaMKII deficiency causes immature dentate gyrus, a novel candidate endophenotype of psychiatric disorders. *Molecular Brain*.

[2] Takao K, Kobayashi K, Hagihara H (2013). Deficiency of Schnurri-2, an MHC enhancer binding protein, induces mild chronic inflammation in the brain and confers molecular, neuronal, and behavioral phenotypes related to schizophrenia. *Neuropsychopharmacology*.

[3] Ohira K, Kobayashi K, Toyama K (2013). Synaptosomal-associated protein 25 mutation converts dentate granule cells to an immature state in adult mice. *Molecular Brain*.

[4] Hagihara H, Nakamura HK, Toyama K, Graef IA, Crabtree GR, Miyakawa T Forebrain-specific calcineurin deficiency causes immaturity of the dentate granule cells in adult mice.

[5] Kobayashi K, Ikeda Y, Sakai A (2010). Reversal of hippocampal neuronal maturation by serotonergic antidepressants. *Proceedings of the National Academy of Sciences of the United States of America*.

[6] Shin R, Kobayashi K, Hagihara H (2013). The immature dentate gyrus represents a common endophenotype of psychiatric disorders and epilepsy. *Bipolar Disorders*.

[7] Walton NM, Zhou Y, Kogan JH (2012). Detection of an immature dentate gyrus feature in human schizophrenia/bipolar patients. *Translational Psychiatry*.

[8] Takao K, Yamasaki N, Miyakawa T (2007). Impact of brain-behavior phenotypying of genetically-engineered mice on research of neuropsychiatric disorders. *Neuroscience Research*.

[9] Gerber DJ, Hall D, Miyakawa T (2003). Evidence for association of schizophrenia with genetic variation in the 8p21.3 gene, PPP3CC, encoding the calcineurin gamma subunit. *Proceedings of the National Academy of Sciences of the United States of America*.

[10] Miyakawa T, Leiter LM, Gerber DJ (2003). Conditional calcineurin knockout mice exhibit multiple abnormal behaviors related to schizophrenia. *Proceedings of the National Academy of Sciences of the United States of America*.

[11] Winder DG, Sweatt JD (2001). Roles of serine/threonine phosphatases in hippocampal synaptic plasticity. *Nature Reviews Neuroscience*.

[12] Shoji H, Hagihara H, Takao K, Hattori S, Miyakawa T (2012). T-maze forced alternation and left-right discrimination tasks for assessing working and reference memory in mice. *Journal of Visualized Experiments*.

[13] Matsuo N, Yamasaki N, Ohira K (2009). Neural activity changes underlying the working memory deficit in alpha-CaMKII heterozygous knockout mice. *Frontiers in Behavioral Neuroscience*.

[14] Young CE, Arima K, Xie J (1998). SNAP-25 deficit and hippocampal connectivity in schizophrenia. *Cerebral Cortex*.

[15] Lewis CM, Levinson DF, Wise LH (2003). Genome scan meta-analysis of schizophrenia and bipolar disorder, part II: Schizophrenia. *American Journal of Human Genetics*.

[16] Barr CL, Feng Y, Wigg K (2000). Identification of DNA variants in the SNAP-25 gene and linkage study of these polymorphisms and attention-deficit hyperactivity disorder. *Molecular Psychiatry*.

[17] Choi TK, Lee HS, Kim JW (2007). Support for the MnlI polymorphism of SNAP25; a Korean ADHD case-control study. *Molecular Psychiatry*.

[18] Zhang Y, Vilaythong AP, Yoshor D, Noebels JL (2004). Elevated thalamic low-voltage-activated currents precede the onset of absence epilepsy in the SNAP25-deficient mouse mutant Coloboma. *The Journal of Neuroscience*.

[19] Kataoka M, Kuwahara R, Matsuo R, Sekiguchi M, Inokuchi K, Takahashi M (2006). Development- and activity-dependent regulation of SNAP-25 phosphorylation in rat brain. *Neuroscience Letters*.

[20] Horiuchi Y, Ishiguro H, Koga M (2007). Support for association of the PPP3CC gene with schizophrenia. *Molecular Psychiatry*.

[21] Zeng H, Chattarji S, Barbarosie M (2001). Forebrain-specific calcineurin knockout selectively impairs bidirectional synaptic plasticity and working/episodic-like memory. *Cell*.

[22] Hagihara H, Toyama K, Yamasaki N, Miyakawa T (2009). Dissection of hippocampal dentate gyrus from adult mouse. *Journal of Visualized Experiments*.

[23] Valor LM, Jancic D, Lujan R, Barco A (2010). Ultrastructural and transcriptional profiling of neuropathological misregulation of CREB function. *Cell Death & Differentiation*.

[24] Merz K, Herold S, Lie DC (2011). CREB in adult neurogenesis—master and partner in the development of adult-born neurons?. *The European Journal of Neuroscience*.

[25] Ohira K, Miyakawa T (2011). Chronic treatment with fluoxetine for more than 6 weeks decreases neurogenesis in the subventricular zone of adult mice. *Molecular Brain*.

[26] Dulawa SC, Holick KA, Gundersen B, Hen R (2004). Effects of chronic fluoxetine in animal models of anxiety and depression. *Neuropsychopharmacology*.

[27] Alonso R, Griebel G, Pavone G, Stemmelin J, Le Fur G, Soubrié P (2004). Blockade of CRF1 or V1B receptors reverses stress-induced suppression of neurogenesis in a mouse model of depression. *Molecular Psychiatry*.

[28] David DJ, Samuels BA, Rainer Q (2009). Neurogenesis-dependent and -independent effects of fluoxetine in an animal model of anxiety/depression. *Neuron*.

[29] Kobayashi K, Ikeda Y, Suzuki H (2011). Behavioral destabilization induced by the selective serotonin reuptake inhibitor fluoxetine. *Molecular Brain*.

[30] Detour J, Schroeder H, Desor D, Nehlig A (2005). A 5-month period of epilepsy impairs spatial memory, decreases anxiety, but spares object recognition in the lithium-pilocarpine model in adult rats. *Epilepsia*.

[31] Sillaber I, Panhuysen M, Henniger MSH (2008). Profiling of behavioral changes and hippocampal gene expression in mice chronically treated with the SSRI paroxetine. *Psychopharmacology*.

[32] Hagihara H, Ohira K, Toyama K, Miyakawa T (2011). Expression of the AMPA receptor subunits GluR1 and GluR2 is associated with granule cell maturation in the dentate gyrus. *Frontiers in Neurogenesis*.

[33] Hagihara H, Hara M, Tsunekawa K, Nakagawa Y, Sawada M, Nakano K (2005). Tonic-clonic seizures induce division of neuronal progenitor cells with concomitant changes in expression of neurotrophic factors in the brain of pilocarpine-treated mice. *Molecular Brain Research*.

[34] Okamoto OK, Janjoppi L, Bonone FM (2010). Whole transcriptome analysis of the hippocampus: toward a molecular portrait of epileptogenesis. *BMC Genomics*.

[35] Cha BH, Akman C, Silveira DC, Liu X, Holmes GL (2004). Spontaneous recurrent seizure following status epilepticus enhances dentate gyrus neurogenesis. *Brain and Development*.

[36] Karpova NN, Pickenhagen A, Lindholm J (2011). Fear erasure in mice requires synergy between antidepressant drugs and extinction training. *Science*.

[37] Hensch TK (2005). Critical period plasticity in local cortical circuits. *Nature Reviews Neuroscience*.

[38] Berretta S (2012). Extracellular matrix abnormalities in schizophrenia. *Neuropharmacology*.

[39] Pizzorusso T, Medini P, Berardi N, Chierzi S, Fawcett JW, Maffei L (2002). Reactivation of ocular dominance plasticity in the adult visual cortex. *Science*.

[40] Gogolla N, Caroni P, Lüthi A, Herry C (2009). Perineuronal nets protect fear memories from erasure. *Science*.

[41] Vetencourt JFM, Sale A, Viegi A (2008). The antidepressant fluoxetine restores plasticity in the adult visual cortex. *Science*.

[42] Lein ES, Hawrylycz MJ, Ao N (2007). Genome-wide atlas of gene expression in the adult mouse brain. *Nature*.

[43] Altar CA, Jurata LW, Charles V (2005). Deficient hippocampal neuron expression of proteasome, ubiquitin, and mitochondrial genes in multiple schizophrenia cohorts. *Biological Psychiatry*.

[44] Hyde TM, Lipska BK, Ali T (2011). Expression of GABA signaling molecules KCC2, NKCC1, and GAD1 in cortical development and schizophrenia. *The Journal of Neuroscience*.

[45] Blaesse P, Airaksinen MS, Rivera C, Kaila K (2009). Cation-chloride cotransporters and neuronal function. *Neuron*.

[46] Lewis DA, Cruz DA, Melchitzky DS, Pierri JN (2001). Lamina-specific deficits in parvalbumin-immunoreactive varicosities in the prefrontal cortex of subjects with schizophrenia: evidence for fewer projections from the thalamus. *The American Journal of Psychiatry*.

[47] Reynolds GP, Zhang ZJ, Beasley CL (2001). Neurochemical correlates of cortical GABAergic deficits in schizophrenia: selective losses of calcium binding protein immunoreactivity. *Brain Research Bulletin*.

[48] Beasley CL, Zhang ZJ, Patten I, Reynolds GP (2002). Selective deficits in prefrontal cortical GABAergic neurons in schizophrenia defined by the presence of calcium-binding proteins. *Biological Psychiatry*.

[49] Beasley CL, Reynolds GP (1997). Parvalbumin-immunoreactive neurons are reduced in the prefrontal cortex of schizophrenics. *Schizophrenia Research*.

[50] Wang AY, Lohmann KM, Yang CK (2011). Bipolar disorder type 1 and schizophrenia are accompanied by decreased density of parvalbumin- and somatostatin-positive interneurons in the parahippocampal region. *Acta Neuropathologica*.

[51] Okaty BW, Miller MN, Sugino K, Hempel CM, Nelson SB (2009). Transcriptional and electrophysiological maturation of neocortical fast-spiking GABAergic interneurons. *The Journal of Neuroscience*.

[52] Gandal MJ, Nesbitt AM, McCurdy RM, Alter MD (2012). Measuring the maturity of the fast-spiking interneuron transcriptional program in autism, schizophrenia, and bipolar disorder. *PLoS ONE*.

[53] Pantazopoulos H, Woo TUW, Lim MP, Lange N, Berretta S (2010). Extracellular matrix-glial abnormalities in the amygdala and entorhinal cortex of subjects diagnosed with schizophrenia. *Archives of General Psychiatry*.

[54] Pantazopoulos H, Lange N, Baldessarini RJ, Berretta S (2007). Parvalbumin neurons in the entorhinal cortex of subjects diagnosed with bipolar disorder or schizophrenia. *Biological Psychiatry*.

[55] Fukuda S, Yamasaki Y, Iwaki T (2002). Characterization of the biological functions of a transcription factor, c-myc intron binding protein 1 (MIBP1). *Journal of Biochemistry*.

[56] Purcell SM, Wray NR, Stone JL (2009). Common polygenic variation contributes to risk of schizophrenia and bipolar disorder. *Nature*.

[57] Shi J, Levinson DF, Duan J (2009). Common variants on chromosome 6p22. 1 are associated with schizophrenia. *Nature*.

[58] Shi Y, Li Z, Xu Q (2011). Common variants on 8p12 and 1q24. 2 confer risk of schizophrenia. *Nature Genetics*.

[59] Stefansson H, Ophoff RA, Steinberg S (2009). Common variants conferring risk of schizophrenia. *Nature*.

[60] Yue WH, Wang H-F, Sun LD (2011). Genome-wide association study identifies a susceptibility locus for schizophrenia in Han Chinese at 11p11. 2. *Nature Genetics*.

[61] van den Elsen PJ, Gobin SJP, van Eggermond MCJA, Peijnenburg A (1998). Regulation of MHC class I and II gene transcription: differences and similarities. *Immunogenetics*.

[62] Kimura MY, Hosokawa H, Yamashita M (2005). Regulation of T helper type 2 cell differentiation by murine Schnurri-2. *Journal of Experimental Medicine*.

[63] Kimura MY, Iwamura C, Suzuki A (2007). Schnurri-2 controls memory Th1 and Th2 cell numbers in vivo. *Journal of Immunology*.

[64] Muller N, Schwarz M (2006). Schizophrenia as an inflammation-mediated dysbalance of glutamatergic neurotransmission. *Neurotoxicity Research*.

[65] Miller BH, Schultz LE, Gulati A, Cameron MD, Pletcher MT (2008). Genetic regulation of behavioral and neuronal responses to fluoxetine. *Neuropsychopharmacology*.

[66] Kataoka M, Yamamori S, Suzuki E (2011). A single amino acid mutation in SNAP-25 induces anxiety-related behavior in mouse. *PLoS ONE*.

[67] Otsuka S, Yamamori S, Watanabe S (2011). PKC-dependent phosphorylation of SNAP-25 plays a crucial role in the suppression of epileptogenesis and anxiety-related behavior in postnatal period of mouse. *Neuroscience Research*.

[68] Vezzani A (2005). Inflammation and epilepsy. *Epilepsy Currents*.

[69] Vezzani A, Granata T (2005). Brain inflammation in epilepsy: experimental and clinical evidence. *Epilepsia*.

[70] Tomkins O, Friedman O, Ivens S (2007). Blood-brain barrier disruption results in delayed functional and structural alterations in the rat neocortex. *Neurobiology of Disease*.

[71] van Vliet EA, da C. Araújo S, Redeker S, Van Schaik R, Aronica E, Gorter JA (2007). Blood-brain barrier leakage may lead to progression of temporal lobe epilepsy. *Brain*.

[72] Uva L, Librizzi L, Marchi N (2008). Acute induction of epileptiform discharges by pilocarpine in the in vitro isolated guinea-pig brain requires enhancement of blood-brain barrier permeability. *Neuroscience*.

[73] Fabene PF, Mora GN, Martinello M (2008). A role for leukocyte-endothelial adhesion mechanisms in epilepsy. *Nature Medicine*.

[74] Zhou M, Li W, Huang S (2013). mTOR inhibition ameliorates cognitive and affective deficits caused by Disc1 knockdown in adult-born dentate granule neurons. *Neuron*.

[75] St Clair D, Blackwood D, Muir W (1990). Association within a family of a balanced autosomal translocation with major mental illness. *The Lancet*.

[76] Burnashev N, Monyer H, Seeburg PH, Sakmann B (1992). Divalent ion permeability of AMPA receptor channels is dominated by the edited form of a single subunit. *Neuron*.

[77] Hollmann M, Hartley M, Heinemann S (1991). Ca2+ permeability of KA-AMPA-gated glutamate receptor channels depends on subunit composition. *Science*.

[78] Hume RI, Dingledine R, Heinemann SF (1991). Identification of a site in glutamate receptor subunits that controls calcium permeability. *Science*.

[79] Zhao C, Deng W, Gage FH (2008). Mechanisms and functional implications of adult neurogenesis. *Cell*.

[80] Jessberger S, Kempermann G (2003). Adult-born hippocampal neurons mature into activity-dependent responsiveness. *The European Journal of Neuroscience*.

[81] Goldman-Rakic PS (1994). Working memory dysfunction in schizophrenia. *Journal of Neuropsychiatry and Clinical Neurosciences*.

[82] Elvevåg B, Goldberg TE (2000). Cognitive impairment in schizophrenia is the core of the disorder. *Critical Reviews in Neurobiology*.

[83] Braff DL, Geyer MA (1990). Sensorimotor gating and schizophrenia: human and animal model studies. *Archives of General Psychiatry*.

[84] American Psychiatric Association (1994). *Diagnostic and Statistical Manual of Mental Disorders*.

[85] Gainetdinov RR, Mohn AR, Bohn LM, Caron MG (2001). Glutamatergic modulation of hyperactivity in mice lacking the dopamine transporter. *Proceedings of the National Academy of Sciences of the United States of America*.

[86] Maycox PR, Kelly F, Taylor A (2009). Analysis of gene expression in two large schizophrenia cohorts identifies multiple changes associated with nerve terminal function. *Molecular Psychiatry*.

[87] Zhang ZJ, Reynolds GP (2002). A selective decrease in the relative density of parvalbumin-immunoreactive neurons in the hippocampus in schizophrenia. *Schizophrenia Research*.

[88] Knable MB, Barci BM, Webster MJ, Meador-Woodruff J, Torrey EF (2004). Molecular abnormalities of the hippocampus in severe psychiatric illness: postmortem findings from the Stanley Neuropathology Consortium. *Molecular Psychiatry*.

[89] Benes FM, Lim B, Matzilevich D, Walsh JP, Subburaju S, Minns M (2007). Regulation of the GABA cell phenotype in hippocampus of schizophrenics and bipolars. *Proceedings of the National Academy of Sciences of the United States of America*.

[90] Pierri JN, Chaudry AS, Woo TUW, Lewis DA (1999). Alterations in chandelier neuron axon terminals in the prefrontal cortex of schizophrenic subjects. *The American Journal of Psychiatry*.

[91] Gallinat J, Winterer G, Herrmann CS, Senkowski D (2004). Reduced oscillatory gamma-band responses in unmedicated schizophrenic patients indicate impaired frontal network processing. *Clinical Neurophysiology*.

[92] Moran LV, Hong LE (2011). High vs low frequency neural oscillations in schizophrenia. *Schizophrenia Bulletin*.

[93] Sponheim SR, Clementz BA, Iacono WG, Beiser M (1994). Resting EEG in first-episode and chronic schizophrenia. *Psychophysiology*.

[94] Abi-Dargham A (2004). Do we still believe in the dopamine hypothesis? New data bring new evidence. *The International Journal of Neuropsychopharmacology*.

[95] Winterer G, Weinberger DR (2004). Genes, dopamine and cortical signal-to-noise ratio in schizophrenia. *Trends in Neuroscience*.

[96] Cousins DA, Butts K, Young AH (2009). The role of dopamine in bipolar disorder. *Bipolar Disorders*.

[97] Novak G, Seeman P (2010). Hyperactive mice show elevated D2High receptors, a model for schizophrenia: calcium/calmodulin-dependent kinase II alpha knockouts. *Synapse*.

[98] Maxwell CR, Kanes SJ, Abel T, Siegel SJ (2004). Phosphodiesterase inhibitors: a novel mechanism for receptor-independent antipsychotic medications. *Neuroscience*.

[99] Kanes SJ, Tokarczyk J, Siegel SJ, Bilker W, Abel T, Kelly MP (2007). Rolipram: a specific phosphodiesterase 4 inhibitor with potential antipsychotic activity. *Neuroscience*.

[100] Lisman J, Schulman H, Cline H (2002). The molecular basis of CaMKII function in synaptic and behavioural memory. *Nature Reviews Neuroscience*.

[101] Weinberger DR (1987). Implications of normal brain development for the pathogenesis of schizophrenia. *Archives of General Psychiatry*.

[102] Weinberger DR, McClure RK (2002). Neurotoxicity, neuroplasticity, and magnetic resonance imaging morphometry: what is happening in the schizophrenic brain?. *Archives of General Psychiatry*.

[103] Marenco S, Weinberger DR (2000). The neurodevelopmental hypothesis of schizophrenia: following a trail of evidence from cradle to grave. *Development and Psychopathology*.

[104] Cannon TD, Rosso IM, Bearden CE, Sanchez LE, Hadley T (1999). A prospective cohort study of neurodevelopmental processes in the genesis and epigenesis of schizophrenia. *Development and Psychopathology*.

[105] Lipska BK (2004). Using animal models to test a neurodevelopmental hypothesis of schizophrenia. *Journal of Psychiatry and Neuroscience*.

[106] Ekdahl CT, Claasen J-H, Bonde S, Kokaia Z, Lindvall O (2003). Inflammation is detrimental for neurogenesis in adult brain. *Proceedings of the National Academy of Sciences of the United States of America*.

[107] Das S, Basu A (2008). Inflammation: a new candidate in modulating adult neurogenesis. *Journal of Neuroscience Research*.

[108] Hunter RL, Dragicevic N, Seifert K (2007). Inflammation induces mitochondrial dysfunction and dopaminergic neurodegeneration in the nigrostriatal system. *Journal of Neurochemistry*.

[109] Fischer MT, Sharma R, Lim JL (2012). NADPH oxidase expression in active multiple sclerosis lesions in relation to oxidative tissue damage and mitochondrial injury. *Brain*.

[110] Pang Y, Cai Z, Rhodes PG (2003). Disturbance of oligodendrocyte development, hypomyelination and white matter injury in the neonatal rat brain after intracerebral injection of lipopolysaccharide. *Developmental Brain Research*.

[111] Bruck W, Pfortner R, Pham T (2012). Reduced astrocytic NF-*κ*B activation by laquinimod protects from cuprizone-induced demyelination. *Acta Neuropathologica*.

[112] Reif A, Fritzen S, Finger M (2006). Neural stem cell proliferation is decreased in schizophrenia, but not in depression. *Molecular Psychiatry*.

[113] Keshavan MS, Nasrallah HA, Tandon R (2011). Schizophrenia, “Just the Facts” 6. Moving ahead with the schizophrenia concept: from the elephant to the mouse. *Schizophrenia Research*.

[114] Nawa H, Takei N (2006). Recent progress in animal modeling of immune inflammatory processes in schizophrenia: implication of specific cytokines. *Neuroscience Research*.

[115] Patterson PH (2009). Immune involvement in schizophrenia and autism: etiology, pathology and animal models. *Behavioural Brain Research*.

[116] Brown AS, Patterson PH (2011). Maternal infection and schizophrenia: implications for prevention. *Schizophrenia Bulletin*.

[117] Hsiao EY, McBride SW, Chow J, Mazmanian SK, Patterson PH (2012). Modeling an autism risk factor in mice leads to permanent immune dysregulation. *Proceedings of the National Academy of Sciences of the United States of America*.

[118] Patterson PH (2002). Maternal infection: window on neuroimmune interactions in fetal brain development and mental illness. *Current Opinion in Neurobiology*.

[119] Shi L, Fatemi SH, Sidwell RW, Patterson PH (2003). Maternal influenza infection causes marked behavioral and pharmacological changes in the offspring. *The Journal of Neuroscience*.

[120] Ioannidis JPA, Ntzani EE, Trikalinos TA, Contopoulos-Ioannidis DG (2001). Replication validity of genetic association studies. *Nature Genetics*.

[121] Ayalew M, Le-Niculescu H, Levey DF (2012). Convergent functional genomics of schizophrenia: from comprehensive understanding to genetic risk prediction. *Molecular Psychiatry*.

[122] Chowdari KV, Mirnics K, Semwal P (2002). Association and linkage analyses of RGS4 polymorphisms in schizophrenia. *Human Molecular Genetics*.

[123] Mirnics K, Middleton FA, Stanwood GD, Lewis DA, Levitt P (2001). Disease-specific changes in regulator of G-protein signaling 4 (RGS4) expression in schizophrenia. *Molecular Psychiatry*.

[124] Petryshen TL, Middleton FA, Tahl AR (2005). Genetic investigation of chromosome 5q GABAA receptor subunit genes in schizophrenia. *Molecular Psychiatry*.

[125] Allen NC, Bagade S, McQueen MB (2008). Systematic meta-analyses and field synopsis of genetic association studies in schizophrenia: the SzGene database. *Nature Genetics*.

[126] Huffaker SJ, Chen J, Nicodemus KK (2009). A primate-specific, brain isoform of KCNH2 affects cortical physiology, cognition, neuronal repolarization and risk of schizophrenia. *Nature Medicine*.

[127] Zakharyan R, Khoyetsyan A, Arakelyan A (2011). Association of C1QB gene polymorphism with schizophrenia in Armenian population. *BMC Medical Genetics*.

[128] Ekelund J, Lichtermann D, Hovatta I (2000). Genome-wide scan for schizophrenia in the Finnish population: evidence for a locus on chromosome 7q22. *Human Molecular Genetics*.

[129] Yan W, Guan X-Y, Green ED (2000). Childhood-onset schizophrenia/autistic disorder and t(1;7) reciprocal translocation: identification of a BAC contig spanning the translocation breakpoint at 7q21. *American Journal of Medical Genetics*.

[130] Bradford M, Law MH, Stewart AD, Shaw DJ, Megson IL, Wei J (2009). The TGM2 gene is associated with schizophrenia in a british population. *American Journal of Medical Genetics B*.

[131] Flynn SW, Lang DJ, Mackay AL (2003). Abnormalities of myelination in schizophrenia detected in vivo with MRI, and post-mortem with analysis of oligodendrocyte proteins. *Molecular Psychiatry*.

[132] Iwamoto K, Bundo M, Kato T (2005). Altered expression of mitochondria-related genes in postmortem brains of patients with bipolar disorder or schizophrenia, as revealed by large-scale DNA microarray analysis. *Human Molecular Genetics*.

[133] Olton DS, Papas BC (1979). Spatial memory and hippocampal function. *Neuropsychologia*.

[134] Goldman-Rakic PS (1996). Regional and cellular fractionation of working memory. *Proceedings of the National Academy of Sciences of the United States of America*.

[135] McLamb RL, Mundy WR, Tilson HA (1988). Intradentate colchicine disrupts the acquisition and performance of a working memory task in the radial arm maze. *NeuroToxicology*.

[136] Emerich DF, Walsh TJ (1989). Selective working memory impairments following intradentate injection of colchicine: attenuation of the behavioral but not the neuropathological effects by gangliosides GM1 and AGF2. *Physiology & Behavior*.

[137] Morris AM, Churchwell JC, Kesner RP, Gilbert PE (2012). Selective lesions of the dentate gyrus produce disruptions in place learning for adjacent spatial locations. *Neurobiology of Learning and Memory*.

[138] Kesner RP (2007). Behavioral functions of the CA3 subregion of the hippocampus. *Learning and Memory*.

[139] Shapiro ML, Olton DS (1994). *Memory Systems 1994*.

[140] Aimone JB, Wiles J, Gage FH (2006). Potential role for adult neurogenesis in the encoding of time in new memories. *Nature Neuroscience*.

[141] Yassa MA, Stark CEL (2011). Pattern separation in the hippocampus. *Trends in Neurosciences*.

[142] Marr D (1971). Simple memory: a theory for archicortex. *Philosophical Transactions of the Royal Society of London B*.

[143] McNaughton BL, Morris RGM (1987). Hippocampal synaptic enhancement and information storage within a distributed memory system. *Trends in Neurosciences*.

[144] O’Reilly RC, McClelland JL (1994). Hippocampal conjunctive encoding, storage, and recall: avoiding a trade-off. *Hippocampus*.

[145] Treves A, Tashiro A, Witter ME, Moser EI (2008). What is the mammalian dentate gyrus good for?. *Neuroscience*.

[146] Gilbert PE, Kesner RP, Lee I (2001). Dissociating hippocampal subregions: a double dissociation between dentate gyrus and CA1. *Hippocampus*.

[147] Gilbert PE, Kesner RP (2003). Localization of function within the dorsal hippocampus: the role of the CA3 subregion in paired-associate learning. *Behavioral Neuroscience*.

[148] Lee I, Kesner RP (2004). Different contributions of dorsal hippocampal subregios to emory acquisation and retrieval in contextual fear-conditioning. *Hippocampus*.

[149] Lee I, Kesner RP (2004). Encoding versus retrieval of spatial memory: double dissociation between the dentate gyrus and the perforant path inputs into CA3 in the dorsal hippocampus. *Hippocampus*.

[150] Leutgeb JK, Leutgeb S, Moser M-B, Moser EI (2007). Pattern separation in the dentate gyrus and CA3 of the hippocampus. *Science*.

[151] McHugh TJ, Jones MW, Quinn JJ (2007). Dentate gyrus NMDA receptors mediate rapid pattern separation in the hippocampal network. *Science*.

[152] Aimone JB, Deng W, Gage FH (2011). Resolving new memories: a critical look at the dentate gyrus, adult neurogenesis, and pattern separation. *Neuron*.

[153] Marín-Burgin A, Mongiat LA, Pardi MB, Schinder AF (2012). Unique processing during a period of high excitation/inhibition balance in adult-born neurons. *Science*.

[154] Nakashiba T, Cushman JD, Pelkey KA (2012). Young dentate granule cells mediate pattern separation, whereas old granule cells facilitate pattern completion. *Cell*.

[155] Swinkels WAM, Kuyk J, van Dyck R, Spinhoven P (2005). Psychiatric comorbidity in epilepsy. *Epilepsy and Behavior*.

[156] Cifelli P, Grace AA (2012). Pilocarpine-induced temporal lobe epilepsy in the rat is associated with increased dopamine neuron activity. *The International Journal of Neuropsychopharmacology*.

[157] Lodge DJ, Grace AA (2011). Hippocampal dysregulation of dopamine system function and the pathophysiology of schizophrenia. *Trends in Pharmacological Sciences*.

[158] Tamminga CA, Southcott S, Sacco C, Wagner AD, Ghose S (2012). Glutamate dysfunction in hippocampus: relevance of dentate gyrus and CA3 signaling. *Schizophrenia Bulletin*.

[159] Lacefield CO, Itskov V, Reardon T, Hen R, Gordon JA (2012). Effects of adult-generated granule cells on coordinated network activity in the dentate gyrus. *Hippocampus*.

[160] Henze DA, Urban NN, Barrionuevo G (2000). The multifarious hippocampal mossy fiber pathway: a review. *Neuroscience*.

[161] Song J, Christian KM, Ming G, Song H (2012). Modification of hippocampal circuitry by adult neurogenesis. *Developmental Neurobiology*.

[162] Grace AA, Floresco SB, Goto Y, Lodge DJ (2007). Regulation of firing of dopaminergic neurons and control of goal-directed behaviors. *Trends in Neurosciences*.

[163] Gottesman II, Gould TD (2003). The endophenotype concept in psychiatry: etymology and strategic intentions. *The American Journal of Psychiatry*.

